# Exercise mimetics: molecular mechanisms, biological and therapeutic effects

**DOI:** 10.1186/s43556-026-00483-8

**Published:** 2026-06-03

**Authors:** Jienan Zhang, Qi Shao, Teng Cheng, Junjian Lin, Yiqin Tang, Haodong Lu, Yue Zhang, Zhongwang Yu, Li Cao

**Affiliations:** Department of Neurobiology, Institute of Neuroscience, Key Laboratory of Molecular Neurobiology of Ministry of Education, Naval Medical University (NMU), Shanghai, China

**Keywords:** Exercise mimetics, Molecular mechanism, Regeneration, Human disease, Clinical translation

## Abstract

Accumulating evidence underscores the therapeutic benefits of exercise in the prevention and treatment of various diseases. However, many patients are unable to engage in sufficient physical training because of frailty, disability, or disease burden. This challenge has stimulated considerable interest in exercise mimetics, which are pharmacological or biological interventions designed to activate exercise-responsive pathways and recapitulate the key adaptive responses elicited by physical activity. In this Review, we outline the conceptual foundations, classification, historical evolution, and biological rationale of exercise mimetics, and organize the field according to its major mechanisms, including neurotrophic signaling, redox regulation, immune modulation, mechanotransduction, vascular coupling, mitochondrial metabolism, epigenetic remodeling, and inter-organ crosstalk. We further examine how these pathways shape normal physiology and development, with an emphasis on metabolic homeostasis, neurodevelopment, cognition, aging, angiogenesis, and inflammation. Building on this framework, we evaluate emerging applications in major human diseases, including cancer, traumatic injury, cardiovascular disorders, neurodegenerative disorders, and inflammatory or immune-mediated conditions, with particular attention to neuroregeneration and systems-level repair. Finally, we assess clinical and translational advances in biomarker-guided diagnosis, personalized treatment, circulating mediators, drug delivery, combination therapy, and clinical trials. Although exercise mimetics offer a scalable and mechanism-informed therapeutic strategy, most evidence to date remains preclinical, and substantial challenges related to pharmacokinetics, safety, target engagement, and patient heterogeneity must be overcome before clinical implementation.

## Introduction

Physical activity remains one of the most broadly protective exposures in human health, with consistent epidemiological evidence linking higher activity levels to reduced risks of cardiovascular disease, cancer, premature mortality, and cognitive decline [1]. Importantly, accumulating evidence indicates that exercise can overcome both intrinsic and extrinsic barriers to central nervous system (CNS) regeneration in adults, including by stimulating neurogenesis, remyelination, glial phenotypic switching, neuroimmune balance, angiogenesis, blood–brain barrier (BBB) repair, and mitochondrial metabolism [2–6]. Exercise is therefore inherently accessible and cost-effective, offering a practical adjunct or alternative to conventional disease prevention and management. Nevertheless, 31.1% of adults worldwide do not meet recommended activity targets, with inactivity disproportionately affecting older individuals and women [7]. This gap is especially consequential in neurological disease, where disability, frailty, pain, and comorbidity disproportionately constrain the populations most likely to benefit from exercise-induced adaptations. In this context, exercise mimetics are conceptualized as complementary tools that harness exercise-responsive biology when the physiological stimulus of training is limited or unattainable.

Exercise mimetics aim to reproduce selected biological effects of physical activity by pharmacologically engaging signaling pathways that are activated during exercise. The most consistently validated exercise-responsive axes include (1) BDNF-TrkB signaling, which engages the MAPK/ERK, PI3K/AKT, and PLCγ pathways [8, 9]; (2) IGF1-AKT-mTOR signaling [10, 11]; and (3) VEGF-linked neurovascular signaling [12, 13]. Beyond these examples, exercise also activates broader metabolic, oxidative stress, immune, and mechanotransduction programs, such as AMPK-PGC1α signaling [14–16], Nrf2-ARE antioxidant signaling [17], JAK-STAT immune signaling [18, 19], and mechanosensitive modules, including integrin-FAK-RhoA/ROCK signaling [20, 21], YAP/TAZ-Hippo signaling [22–24], and Piezo1-calcium signaling [25]. These exercise-responsive pathways provide a mechanistic rationale for defining exercise mimetics as interventions that engage targets demonstrably activated by exercise and produce measurable downstream outputs.

This review aims to clarify what distinguishes the concept of exercise mimetics from broader pharmacology by proposing a systems-informed framework and a pathway hierarchy that connects exercise mimetics to molecular programs, biological functions, and clinically actionable outputs. Guided by this framework, we first establish the conceptual foundations and definitions of exercise mimetics, and then organize the mechanistic evidence across core signaling, epigenetic regulation, and inter-organ crosstalk. We next synthesize their biological effects in normal physiology and development, and evaluate evidence across major disease contexts with particular emphasis on central nervous system disorders. Finally, we discuss the current clinical and translational applications of exercise mimetics, and outline the key limitations and future directions of the field.

## Conceptual foundations and scope of exercise mimetics

### Definition and classification

Exercise mimetics refer to a class of bioactive compounds that can reproduce or amplify the health benefits of physical activity by targeting specific molecular signaling pathways, without requiring direct physical exertion [26]. Unlike physical training itself, which activates a complex and coordinated network of systemic responses across multiple organs, most exercise mimetics aim to engage specific exercise-responsive pathways or signaling mediators in order to replicate the key health-promoting effects of physical activity. Notably, broad-spectrum exercise mimetics such as betaine, a kidney-derived metabolite, may recapitulate the rejuvenating effects of exercise [27].

Exercise mimetics can be classified in multiple ways. One common approach is to group them according to the key signaling pathways they engage, such as AMPK activators, PPAR agonists, and other pathway-directed interventions. A second classification is based on their intrinsic biological nature, including myokines, metabolites, and growth factors that function as endogenous mediators of exercise adaptation and inter-organ communication. Exercise mimetics can also be categorized by the breadth of their mechanistic action. Whereas single-target agents are designed to modulate a defined molecular node, systems-level or multi-target strategies aim to reproduce broader exercise-like physiological states through the coordinated regulation of multiple pathways, tissues, or signaling networks. Together, these classification schemes highlight the biological diversity of exercise mimetics and provide a conceptual framework for evaluating their mechanisms and therapeutic potential.

In this review, exercise mimetics are classified according to the biological effects they exert and are discussed in a logical sequence, beginning with direct neurotrophic programs and progressing through regulatory, environmental, and systemic layers.

### Historical context and emerging relevance

**Fig. 1:**
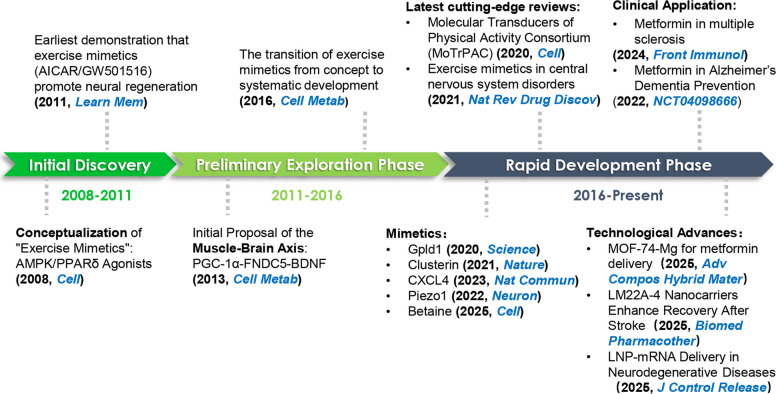
Development milestones of exercise mimetics

Here, we summarize the key milestones that have shaped the developmental trajectory of exercise mimetics (Fig. 1). The seminal study by Narkar et al*.* (2008) demonstrated that pharmacological activation of AMPK and PPARδ could induce training-like adaptations in sedentary animals, thereby establishing the foundation of the field and bringing the concept of exercise mimetics into mainstream biomedical discourse [26]. Subsequent work has expanded the scope of the field by highlighting both the breadth of molecular targets and the diversity of candidate compounds. For example, in 2011, studies revealed that the PPARδ agonist GW501516 and the AMPK activator AICAR improved hippocampal neurogenesis and spatial memory in sedentary mice, providing one of the earliest demonstrations that exercise mimetics can directly promote neural regeneration [28]. By 2013, a study had identified the PGC-1α/FNDC5/BDNF axis as a crucial bridge linking skeletal muscle metabolism to central neuroplasticity [29], further underscoring exercise and its mimetics as systemic regulators of brain health and opening new therapeutic avenues for neurodegenerative diseases. By 2016, comprehensive reviews had systematically catalogued major targets and representative molecules, marking a transition from the conceptual stage toward structured development and therapeutic exploration [30]. Mechanistically, exercise mimetics are not mere ‘on–off switches’ for single pathways but rather modulators of interconnected signaling networks. Their therapeutic targets encompass processes critical for neurorepair, including adult neurogenesis, synaptic remodeling, myelin repair, and axonal regeneration [31]. Recent advances in biomedical technology have further facilitated the discovery and application of exercise mimetics [32]. Integrative omics approaches have delineated the molecular signatures of aerobic adaptation at rest, identifying candidate biomarkers and therapeutic targets such as Gpld1, clusterin, CXCL4, Piezo1, and betaine [31, 33–36]. Advances in gene-editing platforms, bioengineered synthesis, and materials science-enabled delivery are reshaping the discovery-to-dosing continuum for small-molecule exercise mimetics [37–39].

### The biological rationale

Physical exercise elicits coordinated adaptations across multiple organs through evolutionarily conserved molecular and cellular programs that regulate energy balance, stress resistance, tissue remodeling, and neural plasticity. These responses are initiated by changes in cellular energy status, mechanical load, and metabolic flux, which collectively activate a network of signaling pathways including AMPK, PGC-1α, sirtuins, and BDNF-TrkB signaling, among others. Together, these pathways orchestrate mitochondrial biogenesis, synaptic remodeling, angiogenesis, anti-inflammatory responses, and metabolic reprogramming across multiple tissues. Importantly, many of these signaling nodes are biochemically well defined and pharmacologically tractable, providing a mechanistic basis for reproducing selected exercise-induced adaptations through targeted pharmacological interventions.

A second pillar of the biological rationale lies in the systemic nature of exercise signaling. Contracting skeletal muscle acts as an endocrine organ that releases a diverse array of bioactive factors, often termed exerkines, including myokines, metabolites, growth factors, and extracellular vesicles. These circulating mediators transmit exercise signals to distant organs such as the brain, liver, adipose tissue, and vasculature, thereby coordinating whole-body adaptation. For example, muscle-derived factors such as irisin have been implicated in metabolic and neuroimmune regulation. The identification of these circulating mediators suggests that at least part of the exercise response can be reproduced by targeting specific signaling molecules or their downstream pathways.

Finally, the therapeutic rationale for exercise mimetics emerges from the recognition that many individuals who would benefit most from exercise, including patients with neurological disorders, severe injury, advanced age, or frailty, are unable to perform sufficient physical activity to induce robust physiological adaptations. In this context, pharmacological or bioengineered mimetics are conceptualized not as replacements for exercise, but as complementary strategies that engage the core exercise-responsive biological programs when the physiological stimulus of training is limited or unattainable.

Together, these insights provide the conceptual and mechanistic foundation for the development of exercise mimetics aimed at harnessing the health-promoting effects of physical activity.

## Molecular mechanisms of exercise mimetics

### Core signaling pathways and functional outputs

#### Neurotrophic signaling and synaptic plasticity

Among the well-established mediators of exercise-induced neuroregeneration are neurotrophic factors, which consistently sustain neuronal survival, foster synaptic remodeling, and participate in adult hippocampal neurogenesis [40]. Brain-derived neurotrophic factor (BDNF) is the prototypical effector. Brain and serum levels of BDNF are considered reliable markers of cognitive status under both pathological and physiological conditions. Through activation of TrkB receptors, BDNF stimulates the MAPK/ERK, PI3K-AKT, and PLCγ cascades, thereby enhancing dendritic arborization, long-term potentiation (LTP), and learning and memory [8, 9, 41]. Importantly, PI3K-AKT signaling also contributes to the activation of the transcription factor CREB (cAMP response element-binding protein). Activated CREB in turn enhances BDNF transcription and reinforces activity-dependent neurotrophic signaling [42]. This positive regulatory loop is particularly relevant in the context of neuronal regeneration, where the BDNF signaling cascade promotes axonal growth, synaptic remodeling, and the recovery of neural circuits [43, 44]. Exercise reliably elevates BDNF in both the CNS and the periphery [45], whereas BDNF deficiency correlates with amyloid accumulation, tau pathology, and impaired cognition [46]. To pharmacologically reproduce BDNF signaling, several small-molecule mimetics have been developed. 7,8-dihydroxyflavone (7,8-DHF), a small flavonoid compound that acts as a selective TrkB agonist, has been shown to enhance BDNF-like signaling and promote axonal regeneration [47]. In addition, 7,8-DHF protects retinal ganglion cells and promotes axonal regeneration in three-dimensional retinal culture systems and in optic nerve crush (ONC) rat models [47]. Its orally available prodrug, R13, undergoes hepatic conversion to 7,8-DHF, thereby achieving improved bioavailability and sustained receptor engagement. In murine models, R13 administration increases the phosphorylation of TrkB and downstream effectors, including MAPK/ERK1/2 and AKT [48], underscoring its potential as a tractable BDNF mimetic. Accordingly, R13 promoted axon regeneration and functional recovery in mice following sciatic nerve transection [48]. LM22A-4, a small-molecule ligand designed to mimic the loop II domain of BDNF, promotes neurogenesis after hypoxic-ischemic stroke [39, 49]. Moreover, the combined administration of BDNF with ADTC5, a synthetic cadherin peptide that facilitates BDNF transport across the blood–brain barrier, further improved synaptic plasticity and cognitive function [50].

Beyond BDNF, other neurotrophic factors contribute meaningfully to exercise-induced neuroregeneration. Insulin-like growth factor-1 (IGF-1) is a liver- and muscle-derived peptide hormone that rises after exercise, crosses the BBB, and engages AKT/mTOR signaling to stimulate axonal regrowth and oligodendrocyte precursor differentiation [10, 11]. Consistently, combined treadmill exercise and osteopontin supplementation promotes axon regeneration, synaptic reconnection, and motor function recovery through the IGF-1R/Akt/mTOR signaling pathway in mice with C5 cervical crush injury [10].

Vascular endothelial growth factor (VEGF), although best known for its role in angiogenesis, also directly supports hippocampal neurogenesis and synaptic plasticity [51]. Recombinant IGF-1 and VEGF analogues, along with gene therapy vectors, have the potential to replicate these effects.

Collectively, these neurotrophic mimetics provide a direct route to sustain the cell-intrinsic components of exercise biology, including neuronal survival, synaptic efficacy, and activity-dependent neurogenesis, while modern delivery strategies help to overcome BBB and pharmacokinetic constraints.

#### Redox signaling

Exercise activates the Nrf2/ARE pathway, enhancing the activity of antioxidant enzymes such as superoxide dismutase and glutathione peroxidase, which facilitate the clearance of ROS and protect neurons from oxidative damage [17, 52].

Building on this mechanistic link, Nrf2 activators can be explored as pharmacological mimetics of exercise-induced redox adaptations. Dimethyl fumarate (DMF), an FDA-approved fumaric acid ester for multiple sclerosis [53], activates Nrf2 and induces cytoprotective enzymes (e.g., HO-1 and NQO1), which lower oxidative burden and neuroinflammation [54]. Although it does not engage classical endurance-associated circuits such as AMPK or PGC-1α, DMF reproduces a critical dimension of the exercise response: redox homeostasis and resilience to metabolic and inflammatory stressors. Furthermore, DMF exerts pleiotropic effects on mitochondrial integrity and microglial activation through an Nrf2-dependent mechanism [55]. Notably, numerous studies have demonstrated that DMF alleviates cognitive and motor dysfunction by mitigating neurodegeneration, oxidative stress, and neuroinflammation in rodent models of Parkinson’s disease (PD) [56], AD [57, 58], frontotemporal dementia [59], traumatic brain injury (TBI) [60], and ischemia [61]. Moreover, DMF has demonstrated potential benefits in a randomized controlled trial for Friedreich’s ataxia [62]. DMF thus emerges as a candidate exercise mimetic within the redox signaling domain, broadening the therapeutic landscape beyond canonical metabolic mimetics.

#### Immunoregulation

Chronic neuroinflammation is a hallmark barrier to CNS repair, and exercise exerts potent immunomodulatory effects that reshape the neuroinflammatory milieu. Exercise suppresses the release of pro-inflammatory cytokines such as IL-1β and TNF-α, while enhancing the expression of anti-inflammatory mediators including IL-10 and the glycoprotein clusterin [17, 34]. This dual modulation reduces microglial overactivation and reshapes the neuroinflammatory microenvironment, thereby alleviating the inhibitory effects of chronic inflammation on neurogenesis.

Interleukin-6 (IL-6), which is traditionally considered pro-inflammatory, acts as a myokine when released from contracting skeletal muscle. In this context, IL-6 activates metabolic and survival pathways, including PI3K/AKT, MAPK, and AMPK signaling, to support neural repair and metabolic adaptation [63]. Furthermore, IL-6 facilitates glucose uptake and lipid oxidation [64]. Elevated circulating IL-6 has been shown to stimulate the proliferation of postnatal murine forebrain neural stem cells [65], highlighting its context-dependent regenerative potential.

Among exercise-induced humoral mediators, clusterin (CLU) has emerged as a key anti-inflammatory effector. CLU is a liver-derived glycoprotein whose levels are elevated following exercise [34]. A landmark study provided compelling evidence that CLU attenuates cognitive aging and neurodegeneration in a mouse model of AD, corroborated by increased plasma levels of CLU in patients with cognitive impairment following 6 months of exercise [34]. Mechanistically, CLU binds low-density lipoprotein receptor-related protein 8 (LRP8) on brain endothelial cells, suppressing interferon signaling and mitigating neuroinflammation, thereby linking systemic immunity to CNS resilience [34]. In addition, betaine (trimethylglycine), a dietary methyl donor, inhibits TANK-binding kinase 1 (TBK1)-linked inflammatory signaling and reduces cellular senescence across multiple organ systems [36, 66].

Several pharmacological interventions mimic these immune benefits. The AMPK activator metformin and the PPARδ agonist GW501516 suppress microglia-derived cytokines by inhibiting STING and NLRP3 signaling, thereby ameliorating neuroinflammation and attenuating dopaminergic neuronal degeneration and dopamine loss in mouse models of PD [67, 68]. In addition, metformin attenuates senescence and decelerates the aging clock in cynomolgus monkeys, at least partly through the activation of Nrf2 [69]. These agents exemplify how canonical metabolic mimetics extend their impact into the immune dimension of exercise biology.

#### Mechanotransduction and mechanical signaling

The regenerative benefits of exercise are also mediated by mechanotransduction pathways involving cytoskeletal remodeling and mechanical stress sensors. Cyclic mechanical forces generated during exercise (e.g., shear stress and muscle contraction) activate integrin-dependent signaling cascades, including the FAK/RhoA/ROCK axis. These cascades facilitate axonal guidance and synaptic plasticity, thereby providing a molecular foundation for the reconstruction of functional neural connections [20, 21]. In addition, the mechanical properties of the ECM play a unique role in regulating neural stem cell fate. Reduced ECM stiffness supports the directional differentiation of neural stem cells via the YAP/TAZ-Hippo signaling pathway [22–24].

Moreover, mechanosensitive ion channels transduce direct mechanical stimuli. Piezo1, which is expressed in astrocytes, promotes neuronal synaptic maturation and axonal regeneration by initiating calcium influx, modulating ATP release, and enhancing synaptic plasticity [25]. Notably, Piezo1 deficiency results in significantly reduced hippocampal volume and impaired neurogenesis in murine models, underscoring its central role in exercise-induced neural repair [25].

Although mimetics targeting this dimension are less developed, potential strategies include Piezo1 modulators, FAK inhibitors or activators, and biomaterials engineered to mimic ECM mechanics. These approaches underscore that the benefits of exercise are manifested not only in biochemical but also in biophysical dimensions.

#### Vascular and angiogenic signaling

Cerebral perfusion is fundamental to neuronal survival. Exercise enhances vascular function by upregulating endothelial nitric oxide synthase (eNOS) and increasing nitric oxide (NO) bioavailability, which in turn improves cerebrovascular tone. Simultaneously, exercise upregulates the expression of vascular endothelial growth factor (VEGF), driven by hypoxia and mechanical stress. This activates the PI3K/AKT pathway, which promotes angiogenesis and expands the neurovascular niche [12]. In addition, exercise induces cerebral angiogenesis through the lactate receptor HCAR1, which upregulates VEGF expression and integrates systemic metabolism with neurovascular adaptation [13].

Mimetics that replicate these effects include NO boosters and VEGF mimetics. For example, nicotinamide mononucleotide (NMN), an intermediate in the NAD^+^ biosynthesis pathway, restores endothelial angiogenic capacity and enhances neurovascular coupling by increasing endothelial NO-mediated vasodilation, thereby improving spatial working memory in mice [70]. The VEGF mimetic QK peptide (KLTWQELYQLKYKGI) has also been shown to promote both angiogenesis and neurogenesis [71], further emphasizing the potential of targeting vascular signaling for neural regeneration.

#### Mitochondrial metabolism

Neurons rely on efficient bioenergetics to meet the demands of regeneration. Exercise enhances mitochondrial function primarily through the AMPK/PGC-1α/NRF1/2 axis, which stimulates mitochondrial biogenesis, oxidative phosphorylation, and improved neuronal energy metabolism [14, 15]. In parallel, exercise engages the AMPK/SIRT1 pathway to improve glucose utilization, reduce Aβ deposition, and delay the neurodegeneration associated with Alzheimer’s disease [72].

Several exercise mimetics target these mitochondrial regulatory pathways. AICAR and metformin, both activators of AMPK signaling, reproduce key aspects of exercise-induced metabolic adaptation and have been shown to improve recognition memory and neuroplasticity [73, 74]. AICAR, an AMP analog phosphorylated to ZMP by adenosine kinase, increases mitochondrial content and capacity [75]. In sedentary mice, 4 weeks of AICAR treatment alone enhanced running endurance by 44% [26]. Metformin, a widely used biguanide capable of crossing the BBB, activates AMPK signaling through inhibition of mitochondrial complex I, thereby increasing the AMP/ATP ratio [76, 77]. Through AMPK-dependent mechanisms, metformin attenuates oxidative stress and apoptosis, modulates neuroinflammation and microglial reactivity, and enhances BDNF-associated neuroplastic signaling [78, 79].

Additional compounds influence mitochondrial regulation through PGC-1α-related signaling pathways. For example, AdipoRon, an AdipoR1 agonist, restores mitochondrial dynamics by activating AMPK/SIRT3/PGC-1α signaling, mitigating tau pathology, preserving dendritic integrity, and supporting neurogenesis in models of Alzheimer’s disease and intracerebral hemorrhage [80, 81]. Similarly, PPARδ agonists (e.g., GW501516 and GW0742), PPARγ agonists (e.g., rosiglitazone and pioglitazone), and the dual PPARδ/γ agonist T3D-959 enhance lipid oxidation and mitochondrial efficiency, promote neurite outgrowth, and mitigate neurological disorders, thereby linking peripheral endurance metabolism to central plasticity [26, 82–84]. Bezafibrate, a pan-PPAR agonist, has been shown to enhance mitochondrial function and oxidative phosphorylation capacity, as well as to alleviate amyloid pathology and neuronal loss, thereby improving cognitive and memory function [85].

Beyond AMPK and PGC-1α signaling pathways, exercise engages cAMP/CREB/BDNF signaling to enhance mitochondrial resilience and synaptic plasticity. Forskolin, a diterpene adenylyl cyclase activator, elevates cAMP and stimulates CREB-driven transcription, promoting mitochondrial electron transport chain recovery, remyelination, and neurotrophic support, while also exerting anti-inflammatory and antioxidant effects [86]. In mouse hepatocytes, forskolin stimulates Rpn6 phosphorylation and enhances the degradative capability of the 26S proteasome, thereby improving the elimination of damaged and misregulated proteins [87].

Mitochondria-targeted antioxidants may also serve as valuable adjuncts to exercise mimetics owing to their ability to preserve cellular redox homeostasis and support bioenergetic function. MitoQ, a ubiquinone derivative conjugated to a lipophilic triphenylphosphonium cation, accumulates within the mitochondrial matrix where it reduces oxidative stress and prevents mitochondrial DNA damage. Previous studies have shown that MitoQ exerts protective effects on neurons and reduces axonal inflammation and oxidative stress in several neurodegenerative diseases, including Alzheimer’s disease and Parkinson’s disease [88]. Moreover, supplementation with MitoQ improves exercise performance in middle-aged trained men [89]. It also helps maintain muscle health and physical function during aging [90], does not affect redox responses to acute exercise in older skeletal muscle [91], and enhances training-induced peak power improvements in untrained middle-aged men [92].

Together, mimetics that modulate mitochondrial metabolism, including AMPK activators (e.g., AICAR and metformin), AdipoRon, PPAR agonists (e.g., GW501516, GW0742, rosiglitazone, pioglitazone, T3D-959, and bezafibrate), cAMP/CREB activators (e.g., forskolin), and mitochondrial protectants (e.g., MitoQ), support the energy-intensive processes of neuronal repair.

### Epigenetic modifications and chromatin remodeling

Exercise induces durable epigenetic modifications that stabilize pro-regenerative gene expression. In the hippocampus, exercise leads to CpG demethylation of the *Bdnf* promoter, enhanced histone H3 acetylation, and downregulation of Hdac5, thereby sustaining BDNF transcription [93]. These changes represent a molecular memory of activity, ensuring persistent support for synaptic plasticity and neurogenesis [94].

Exercise also remodels the microRNA (miRNA) landscape of the brain. Voluntary running upregulates miR-21, miR-92a, miR-874, miR-223-3p, miR-129-5p [95], miR-204 [96], miR-29a [97], miR-200a-3p [98], and miR-124-3p [99], while downregulating miR-138, let-7c, and miR-124, which could be involved in regenerative programs after injury [100]. Notably, miR-132 exerts significant effects on exercise-induced adult neurogenesis [101, 102]. MicroRNA-directed tools such as miRNA mimics (chemically stabilized synthetic RNAs that reproduce endogenous miRNA function) and antagomirs (chemically modified antisense oligonucleotides that inhibit miRNA function) have been employed to manipulate exercise-responsive miRNAs. For example, administration of synthetic miR-132 has been reported to enhance neurogenesis in an AD mouse model, partly by reducing Aβ burden and tau phosphorylation through repression of the inositol 1,4,5-trisphosphate 3-kinase B (ITPKB)-BACE1 pathway [103]. Conversely, administration of anti-miR-132 oligonucleotides abolishes the exercise-induced benefits on neurogenesis and cognition in human NSCs and in the *App*^*NL−G−F*^ AD mouse model [103, 104]. In addition, miR-223-3p has been shown to reduce hippocampal neuronal injury and ameliorate anxiety- and depression-like behaviors through inhibition of NLRP3 signaling [105], while miR-129-5p mitigates the cognitive impairment by inhibiting neuroinflammation and maintaining astrocytic glutamate uptake in a frontotemporal dementia model [106]. Hypoxia causes HIF-1α-dependent suppression of miR-204, which indirectly contributes to the post-transcriptional upregulation of VASP and thereby promotes the invasiveness and metastasis of hepatocellular carcinoma [96]. Notably, exercise has been reported to promote glycolysis through the induction of miR-204 [107], suggesting that miR-204 may function as a tumor suppressor. In addition, miR-29a has also been reported to play an epigenetic regulatory role in CNS diseases. Neuronal miR-29a directly binds and inhibits Nras, thereby protecting against obesity via the PI3K-Akt-mTOR pathway in adult mice [108], and ameliorates TBI by directly inhibiting NLRP3 [109]. Moreover, muscle-derived exosomal miR-200a-3p improved memory function in mice with type 2 diabetes mellitus [98], whereas microglial exosomal miR-124-3p alleviated neurodegeneration and improved cognition after repetitive mild TBI via the Rela/ApoE pathway in mice [99]. Collectively, these microRNAs hold promise as exercise mimetics for the treatment of neurological disorders.

As the universal methyl donor, S‑adenosylmethionine (SAM) underpins DNA and histone methylation, shaping gene expression and thereby influencing protein synthesis and cell fate determination [110]. Beyond serving as a biochemical cofactor, SAM is closely linked to nervous system development, contributing to neuronal differentiation and maturation and regulating the synthesis and metabolism of neurotransmitters [111]. Clinical and preclinical studies highlight its relevance to exercise biology. A six-month structured exercise program in older adults with mild cognitive impairment significantly altered genome-wide methylation patterns [112]. Supplementation with SAM in transgenic mouse models reduces amyloid-β and phosphorylated tau accumulation, attenuates neuronal loss, elevates hippocampal BDNF levels, and suppresses microglial activation and pro-inflammatory cytokine release [113, 114]. Conversely, SAM depletion induces H3K4me1 hypomethylation, leading to reduced expression of Slc1a2 and Glu1, and ultimately exacerbating neuroexcitotoxicity in PD [113, 115]. Of note, FLAV-27, a competitive inhibitor of SAM, reduces amyloid-β (Aβ) and p-tau aggregation and rescues cognitive impairment in AD mouse models via H3K9me2/H3K18me-mediated repression [116]. This underscores the importance of maintaining appropriate SAM levels in clinical settings.

These findings highlight that epigenetic mimetics, including microRNAs and SAM, can convert transient activity into enduring transcriptional reprogramming, thereby stabilizing the regenerative benefits of exercise.

### Inter-organ crosstalk

Systemically, exercise orchestrates inter-organ communication that shapes the regenerative capacity of the CNS. Multiple peripheral tissues release circulating factors during exercise that act on the brain to regulate neurogenesis, synaptic plasticity, and neuroinflammation. These signaling networks form several major communication axes, including the muscle-brain, liver-brain, and gut-brain axes, which collectively integrate metabolic, immune, and endocrine signals to support neural repair.

The muscle-brain axis represents one of the best-characterized exercise-responsive signaling systems. Contracting skeletal muscle releases a variety of myokines that influence brain function. Among these, irisin, a cleaved ectodomain of the membrane protein FNDC5, binds αV/β5 integrin receptors in multiple tissues including the brain [117, 118]. Experimental studies suggest that irisin can increase hippocampal BDNF expression through Wnt/β-catenin signaling and improve synaptic plasticity and memory in models of Alzheimer’s disease [119, 120]. These findings have raised the possibility that irisin-related pathways may represent therapeutic targets for metabolic and neurodegenerative disorders [121]. Similarly, lactate, a metabolite released from exercising muscle, functions as a signaling molecule in the brain. Lactate has been reported to enhance learning and memory through SIRT1-dependent activation of the PGC-1α/FNDC5 pathway [122]. This highlights the role of lactate not only as a metabolic byproduct but also as a signaling molecule in the brain, linking exercise-induced metabolic changes to cognitive function. Cathepsin B (CTSB), a lysosomal cysteine protease released by contracting muscle, crosses the BBB and accumulates in the hippocampus and prefrontal cortex, supporting neurogenesis and cognitive function [123]. The application of recombinant CTSB has been shown to increase the expression of BDNF and doublecortin in adult hippocampal progenitor cells, thereby enhancing neurogenesis [124]. Additionally, β-aminoisobutyric acid (BAIBA), another muscle-derived factor, activates the AMPK and PI3K/AKT pathways and has been associated with antioxidant and anti-inflammatory neuroprotective effects [125, 126].

The liver-brain axis represents another critical pathway through which exercise modulates brain function. Exercise promotes the hepatic release of β-hydroxybutyrate (BHB), a ketone produced during fatty-acid oxidation in the liver, which crosses the BBB to enhance BDNF expression, brain energy metabolism, and oligodendrocyte differentiation, ultimately supporting myelin regeneration [127, 128]. Additionally, hepatic glycosylphosphatidylinositol-specific phospholipase D1 (Gpld1) elevates circulating glycosylphosphatidylinositol levels by altering GPI-anchored substrate cleavage, thereby enhancing hippocampal neurogenesis and cognitive function, particularly in aging models [32]. However, in a recent observational study, no clear relationship was observed between GPLD1 and cognitive function in older adults, regardless of physical exercise status [129]. Selenoprotein P (SEPP1), the principal selenium transport protein synthesized in hepatocytes, signals via LRP8 on neural cells to promote NPC proliferation and protect cognition during aging [130]. Furthermore, FGF21, another hepatokine modulated by exercise, has been shown to increase hippocampal synaptic plasticity, reduce systemic inflammation and neuronal cell death, and enhance cognitive function [131].

The gut-brain axis involves bidirectional communication between the gastrointestinal tract and the CNS, with modulation of the gut microbiota playing a critical regulatory role. Prolonged exercise beneficially alters the composition of the gut microbiota, enhancing intestinal barrier function, reducing lipopolysaccharide translocation, and alleviating neuroinflammation associated with neurodegenerative diseases such as AD [132]. Regular moderate exercise has been shown to reduce harmful microbial taxa while increasing the growth of beneficial strains, thus promoting gut health in the elderly [133]. Short-chain fatty acids (SCFAs) such as acetate, propionate, and butyrate, which are produced by the gut microbiome, have been shown to influence neuronal signaling pathways, for example by modulating cFos expression and sympathetic activity in experimental models [134]. Interventions that increase SCFA production, including probiotics and prebiotics, may therefore act as ecosystem-level exercise mimetics, potentially recapitulating some of the systemic benefits of physical activity.

**Fig. 2:**
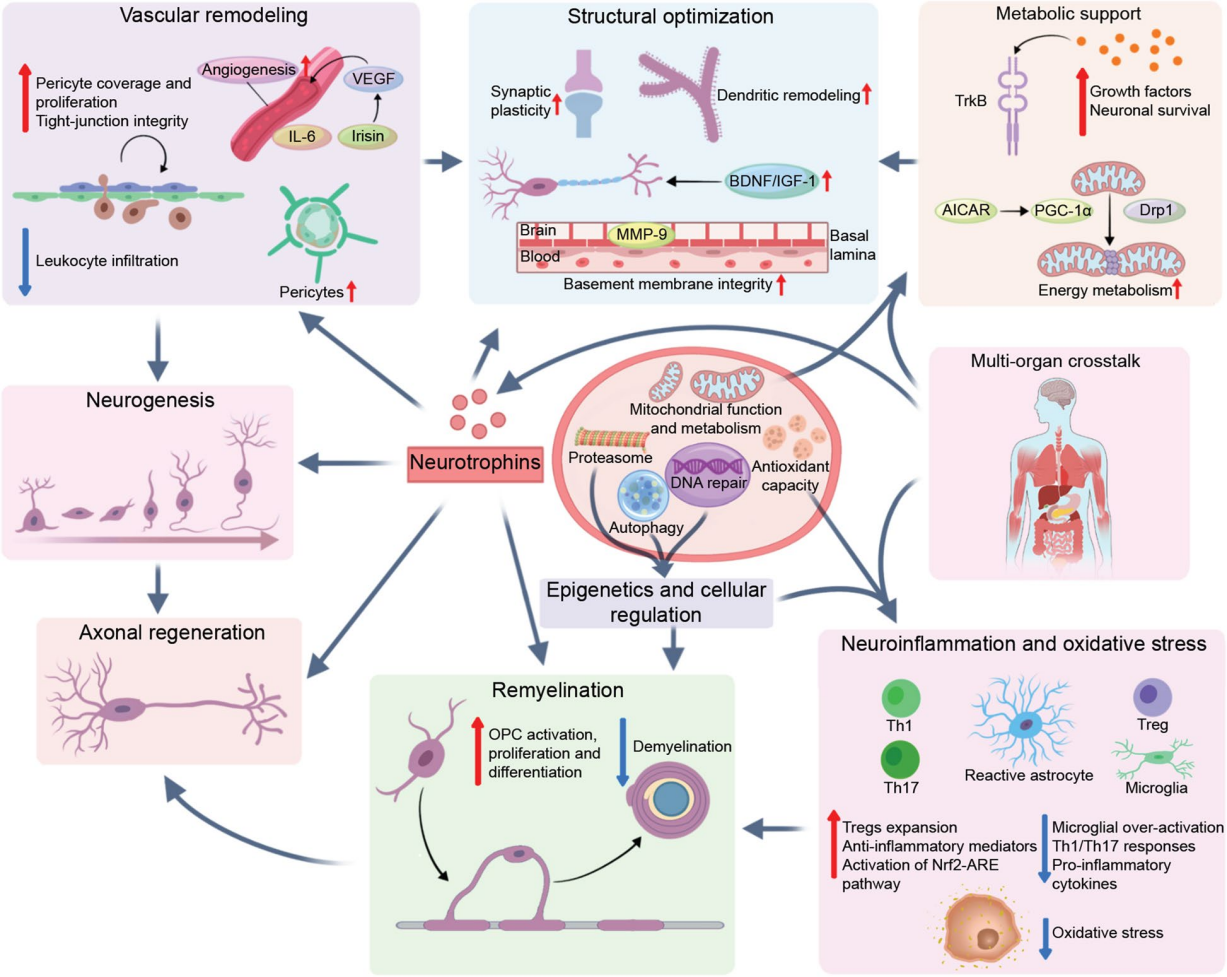
Multi-scale conceptual framework for the benefits of exercise and exercise mimetics. Exercise and exercise mimetics promote central nervous system (CNS) regeneration through interconnected biological programs operating across multiple biological scales. This conceptual framework illustrates how molecular and subcellular mechanisms converge to drive tissue- and system-level repair processes. At the molecular and subcellular level, exercise and exercise mimetics activate core cellular programs that enhance neuronal resilience and regenerative capacity. These include enhanced neurotrophic signaling, improved mitochondrial function and metabolic regulation, strengthened antioxidant defense, activation of DNA repair mechanisms, epigenetic remodeling (such as DNA methylation and histone modifications), and reinforced protein and organelle quality-control systems, including autophagy, lysosomal pathways, and the ubiquitin–proteasome system. Together, these processes restore cellular homeostasis and maintain proteostasis in aging or injured neural tissue. These molecular programs subsequently influence processes at the organ, tissue, and cellular levels. Elevated neurotrophic signaling promotes vascular remodeling, neurogenesis, axonal regeneration, and remyelination, thereby supporting the structural and functional recovery of neural circuits. Metabolic support, largely driven by mitochondrial optimization, further contributes to structural remodeling and neuronal plasticity, while vascular remodeling facilitates neurogenesis by improving the neurogenic niche. In addition, cellular regulatory systems, including DNA repair, epigenetic regulation, and proteostasis pathways, modulate neuroinflammation and oxidative stress, which in turn influence remyelination and neural repair processes. Importantly, multi-organ crosstalk represents an additional regulatory axis. Circulating exercise-induced factors derived from peripheral organs, including muscle, liver, gut, and platelets, can modulate neurotrophic signaling, neuroinflammatory states, and metabolic support within the CNS. Collectively, this framework emphasizes that exercise mimetics do not act on isolated pathways but rather engage interconnected neuro-immune-vascular-metabolic networks, highlighting the systems-level complexity underlying exercise-induced neuroregeneration

In summary, these findings underscore that the regenerative benefits of exercise are inherently systemic and can be harnessed by mimetics targeting peripheral organs. The mechanistic pathways and supporting evidence from experimental models and human studies for representative exercise mimetics are summarized in Table 1. Precision exercise mimetics highlight the potential to deconstruct the pleiotropic benefits of physical activity into discrete, pharmacologically tractable modules that converge on neuroregenerative pathways. By selectively engaging trophic, epigenetic, redox, immune, mechanosensory, vascular, and mitochondrial programs, these agents offer a means of extending the benefits of exercise to individuals who are unable to participate in sufficient physical training (Fig. 2) [135]. As a conceptual bridge between systems physiology and therapeutics, exercise mimetics exemplify a strategy not to replace exercise but rather to harness its most relevant biological signals for targeted neurorepair.

**Table 1:** The classification and mechanistic pathways of exercise mimetics

Classification	Exercise mimetics	Target pathway/functions	Evidence
Neurotrophins	BDNF, 7,8-DHF, R13, LM22A-4	MAPK/ERK, PI3K-AKT, and PLCγ cascades	Mouse model of peripheral nerve injury [48]; Sprague Dawley (SD) rat model of optic nerve injury and retinal ganglion cell degeneration [47]; mouse model of hypoxic-ischemic stroke [39, 49]; middle-aged recreationally trained men [89];
	IGF-1	AKT/mTOR	Mouse model of central nervous system axonal injury [10]; mouse model of myocardial infarction-induced skeletal muscle atrophy [136]; human exercise intervention studies in healthy, obese, and cancer populations [137]
Redox signaling	DMF Metformin	Nrf/ARE	Mouse models of neurodegenerative and demyelinating diseases [55–61]; cynomolgus monkey model [69]
Immunoregulation	IL-6	PI3K/AKT, MAPK, AMPK	Sedentary aged male mice [6, 65, 138]; patients with autoimmune diseases [139]
	CLU	LRP8 mediated interferon signaling	Plasma from exercising male mice transferred into young non-exercising littermates [34]
	Metformin	STING and NLRP3	Obese mice exhibiting hypothalamic aging [68]
	Betaine	TBK1	Aging human and mouse [140]
Mechanostransduction	Piezo1	Initiates calcium influx/p38MAPK-YAP1 pathway and Ccl2-Lcn2 inflammatory autocrine loop	Mouse astrocytes and neurons [25]; mouse model of myocardial infarction [141]; mouse model of osteoporosis [142]
Vascular and angiogenic signaling	nicotinamide mononucleotide (NMN)	Increases endothelial NO-mediated vasodilation, activates sirtuin deacylases	Aged mice with impaired brain microvascular endothelial function and neurovascular coupling [70, 143]
	VEGF mimetic QK peptide	PI3K/AKT	Mouse models [144]
Mitochondrial metabolism	AICAR	AMPK	Mouse skeletal muscle model [26, 145]
	Metformin	AMPK/BDNF/PI3K; improves glucose metabolism	D-galactose inducedrat model of aging-associated neurocognitive impairment [78]; exercise–metformin intervention study in patients with prediabetes and type 2 diabetes mellitus [146]; adults aged 50 years or older with peripheral artery disease [147]
	AdipoRon	AMPK/SIRT3/PGC-1α	Mouse model of intracerebral hemorrhage [81]; mouse model of Alzheimer’s disease [80]; mouse model targeting basolateral amygdala [148]
	GW501516	NLRP3 and PPARß/δ agonist	MPTP-induced mouse Parkinson’s disease model [67]; mouse metabolic model [149]; mouse inflammation model [150]. Mouse tumor-related pathway model [151]
	GW0742	PPARδ/β agonist	Rat model of hypoxic-ischemic [152]; rat model of spinal cord injury [153]; mouse cardiac model [154]
	Rosiglitazone	PPARγ agonist	Mouse N2A cell model [83]
	Pioglitazone	PPARγ agonist	Mouse model of Alzheimer’s disease [84, 155]
	T3D-959	PPARδ/γ agonist	Mild to moderate Alzheimer’s disease patients [156]
	Bezafibrate	Pan-PPAR agonist	Mouse model of Alzheimer’s disease [85]
	Forskolin	cAMP/CREB	Cardiomyopathic hamsters [157]
	MitoQ	Reduces oxidative stress	Mouse model of experimental autoimmune encephalomyelitis [88]; older mouse skeletal muscle model [91]; untrained middle-aged mouse model [92]; older individuals [90]
Epigenetic regulation	miR-135a-5p	Reduces Aβ burden and tau phosphorylation	Mouse model of Alzheimer’s disease [103]
	SAM	Reduces Aβ burden and tau phosphorylation	D-galactose-induced brain aging rat model [114]; 3xTg-AD transgenic mouse [113]
	miR-223-3p	Inhibits the TLR4/MyD88-NF-κB signaling pathway; attenuates hippocampal neuroinflammation	Exercising mice [95]
	miR-129-5p	Alleviates neuroinflammation and improves cognitive function; mediates exercise-induced anti-inflammatory and neuroprotective effects	Exercising mice [95]
	miR-204	Enhances skeletal muscle glycolysis	Acute/chronic treadmill exercise mouse model [107]; miR-204 mimic intravenous injection mouse model [107]
	miR-29a-3p	AMPK, PI3K-Akt, mTOR	Systemic miR-29a/b1 cluster knockout (miR-29KO) male mice [97]
	miR-200a-3p	Regulates the KEAP1/HSP90 signaling pathway; downregulates hippocampal Keap1 mRNA levels and upregulates hippocampal Hsp90aa1 mRNA levels; Upregulates hippocampal Mct2 mRNA levels to improve lactate transport function	Type 2 diabetes mellitus (T2DM) mouse model (ob/ob mice) [98]
	miR-124-3p	Regulates Rela/ApoE signaling pathway; reduces Aβ production; alleviates neurodegeneration; improves cognitive function	Repetitive mild traumatic brain injury (rmTBI) mouse model [99]
Muscle-brain axis	Irisin/FNDC5	Wnt/β-catenin	Mouse model of Alzheimer’s disease [119, 120]; ovariectomy-induced osteolysis mouse model [117]; white adipocyte model including mice and humans [158]
	Lactate	PGC1α/FNDC5/BDNF/GPR81/FARP1 signaling	Male mouse model [122] ; diet-induced obese mouse model and human and racehorse models [159]; marathon runners [160]
	CTSB	Increases BDNF	Mouse and human models [124, 161]
	BAIBA	AMPK and PI3K/AKT	Rat PC12 cell model [125]; Mouse and human models [125]
Liver-brain axis	BHB	Increases BDNF and brain energy metabolism	CPZ-induced mouse model [128]; multiple sclerosis mouse model [162]; high salt-fed hypertensive rat model [163]
	Gpld1	Increases circulating glycosylphosphatidylinositol levels	Aging mouse model [32, 129]; mouse model of antiviral immunity [164]
	SEPP1	LRP8	Mouse model with hippocampal damage and aging [130]
	FGF21	Reduces systemic inflammation and neuronal cell death	Diabetic cardiomyopathy mouse model [165]; human thermogenesis model [166]
Gut-brain axis	SCFAs	Modulates cFos expression; anti-inflammatory	Mouse model [134];human model [167]; 6-week human exercise intervention model [168]

## Biological effects in normal physiology and development

Exercise confers broad benefits on physical health and plays a well-established role in preventing the onset and progression of disease. However, a substantial proportion of individuals fail to attain the optimal amount of exercise owing to time constraints, occupational demands, and lifestyle factors. Exercise mimetics can provide physiological benefits by recapitulating the adaptive responses of skeletal muscle observed during endurance training, thereby helping to maintain or improve overall health. The following sections discuss the roles and mechanisms of exercise and exercise mimetics in key physiological and developmental processes.

### Metabolic homeostasis and systemic health

Maintenance of energy metabolism homeostasis is fundamental to life. Regular exercise represents the most effective intervention for improving metabolic flexibility and energy metabolism. The metabolic benefits of exercise are primarily mediated by mitochondrial remodeling in skeletal muscle and the regulation of cellular energy metabolism.

**Fig. 3:**
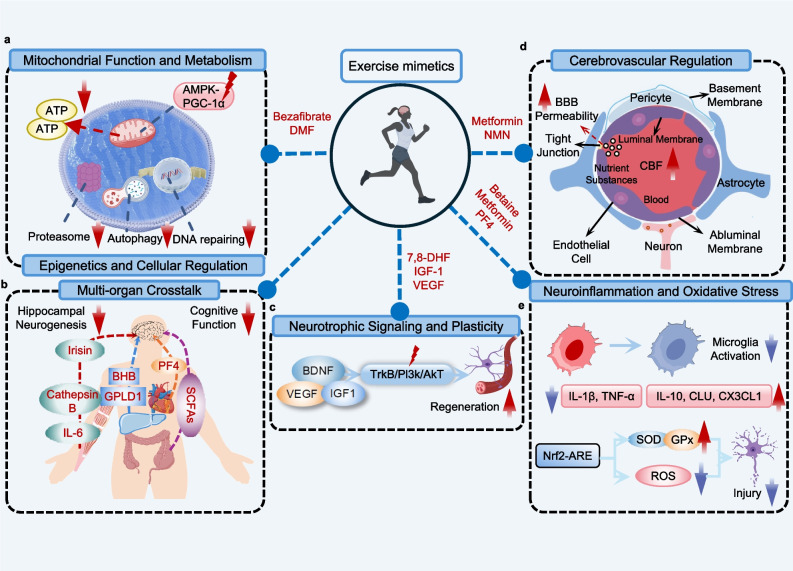
Exercise- and exercise mimetic-induced mechanisms underlying metabolic homeostasis and systemic health. **a** Exercise enhances mitochondrial function and ATP production via the AMPK-PGC-1α pathway while augmenting autophagy and proteostasis; mimetics (e.g., bezafibrate and DMF) restore mitochondrial health in aging. **b** Cross-organ factors (irisin, IL-6, cathepsin B, FGF21, BHB, Gpld1, PF4, and SCFAs) signal to the brain, promoting hippocampal neurogenesis and cognitive resilience. **c** Exercise enhances neurogenesis and plasticity via BDNF, IGF-1, VEGF, and TrkB-PI3K-Akt signaling; mimetics such as 7,8-DHF, IGF-1 gene therapy, and VEGF preconditioning replicate these pathways. **d** Exercise improves cerebral blood flow (via eNOS activation) and BBB integrity through endothelial junctions and pericyte-astrocyte interactions; mimetics such as NMN and metformin reproduce these effects. **e** Exercise suppresses microglial activation, decreases IL-1β/TNF-α, increases IL-10/CLU/CX3CL1, and activates Nrf2-ARE/SOD/GPx, thereby limiting ROS-mediated damage; mimetics (e.g., betaine, metformin, and PF4) provide similar protection

First, exercise improves metabolism and mitochondrial function in peripheral tissues such as muscle, exerting systemic effects that can enhance the function of distant organs, including the brain. Exercise enhances mitochondrial function and metabolism to maintain neuronal energy homeostasis (Fig. 3a). It activates the AMPK-PGC-1α axis to enhance mitochondrial biogenesis and oxidative phosphorylation, thereby increasing ATP production [169]. Guided by these mechanistic insights, pharmacological interventions have been explored as exercise mimetics to reinforce mitochondrial health. The pan-PPAR agonist bezafibrate induces PGC-1α-dependent programs that enhance mitochondrial fatty acid oxidation, oxidative phosphorylation, and mitochondrial biogenesis [170]. Trimetazidine upregulates the expression of PGC-1α, thereby promoting mitochondrial biogenesis and improving energy metabolism [171]. The Nrf2/ARE activator DMF reduces oxidative damage to mitochondrial DNA and enhances mitochondrial biogenesis, mitophagy, and antioxidant defense in the hippocampus of 15-month-old mice, thereby improving short- and long-term memory [172].

Second, exercise induces significant skeletal muscle remodeling, enhancing metabolic capacity and enabling the efficient utilization of glucose and fatty acids as energy sources [173, 174]. Exercise mimetics can directly optimize the utilization of systemic energy substrates, including glucose and lipids. Specifically, exercise mimetics improve systemic energy substrate utilization through activation of the AMPK/PPARδ pathway, increasing resting metabolic rate and preventing excessive fat accumulation. For instance, the AMPK activator AICAR directly promotes muscle glucose uptake and fatty acid oxidation [175], while SLU-PP-33 significantly enhances skeletal muscle energy expenditure and fatty acid oxidation [176].

Third, exercise mimetics can mimic myokines secreted by skeletal muscle, thereby mediating crosstalk among adipose tissue, liver, pancreas, gut, and other important organs. Exogenous β-hydroxybutyrate supplementation can replicate exercise-triggered endogenous ketogenesis while achieving comparable hepatoprotective outcomes [177]. BDNF, acting as an exercise mimetic, targets pancreatic β-cells, thereby enhancing insulin secretion and regulating whole-body glucose homeostasis [178]. Furthermore, irisin, functioning as an exercise mimetic, plays a pivotal role in the regulation of gut metabolism. Depletion of irisin diminishes intestinal microbial diversity, and exogenous irisin supplementation significantly improves the pathological manifestations of microbiota dysbiosis [179].

Crucially, exercise activates cross-organ signaling networks to support brain health (Fig. 3b). Liver-derived metabolites such as BHB improve mitochondrial efficiency and hippocampal plasticity through the liver-brain axis [180]. In addition, exercise-induced modulation of the gut microbiota increases SCFA production, which attenuates microglial activation via the vagus nerve or the circulatory system [181]. Platelet-derived factors such as platelet factor 4 (PF4) also contribute to enhanced hippocampal neurogenesis and cognitive performance during aging [182]. Overexpression of liver-derived Gpld1 can improve hippocampal neurogenesis and cognitive performance in aged rodents [32], suggesting that Gpld1 has the potential to restore neuronal plasticity and function in brain disorders.

These results indicate that exercise preserves systemic homeostasis and health via metabolic and inter-organ communication. Targeted mimetics may replicate these protective effects, providing novel translational approaches for metabolic health, brain function, and the prevention of multi-organ disease.

### Neurodevelopment and cognitive function

Exercise-induced cognitive enhancement operates through the regulation of neurodevelopmental programs and the promotion of neuroplasticity. Integration of animal model data and human neuroimaging evidence reveals hierarchical critical periods along the sensorimotor-association cortical axis during neurodevelopment, with neural plasticity serving as the core driver of neural development [183].

Exercise enhances neuroplasticity by upregulating key neurotrophic factors, including BDNF, IGF-1, and VEGF. These factors activate downstream pathways such as TrkB/PI3K/AKT and Wnt/β-catenin, thereby promoting NSC proliferation, neuronal survival, synaptic remodeling, and angiogenesis [144, 184] (Fig. 3c).

Moreover, the myokine-mediated pathway from skeletal muscle to the brain facilitates cognitive enhancement through the regulation of adult hippocampal neurogenesis and synaptic plasticity, with older adults demonstrating the most significant therapeutic gains [32, 185]. The exercise mimetic AICAR also enhances hippocampal neurogenesis, thereby improving cognitive and motor performance in mice [28, 74]. Muscle-derived myokines such as IL-6, cathepsin B, and irisin influence astrocyte function and promote neurogenesis through pathways such as Wnt/β-catenin [181, 186, 187]. Similarly, akin to exercise-induced lactate, irisin modulates the expression of genes involved in synaptic and neural plasticity, ultimately improving cognitive performance [188].

These findings indicate that, although exercise orchestrates a broad neurotrophic program, targeted mimetics can selectively reproduce elements of this response, highlighting their potential as adjunctive strategies to counteract cognitive decline and as enhancers of cognitive performance.

### Aging and longevity

Aging is characterized by progressive functional decline and multi-organ dysfunction [189]. Accumulating evidence from animal models and human studies indicates that physical exercise can counteract these age-related changes through multifaceted mechanisms. Exercise mimetics provide a translational route for delivering similar benefits to adults who cannot reliably engage in sustained training [190, 191].

First, the excessive accumulation of reactive oxygen species (ROS) during aging constitutes a central driver of organismal senescence. ROS induce tissue damage through multiple mechanisms, including mitochondrial dysfunction, disruption of epigenetic regulatory processes, and oxidative stress. Conventional antioxidants, such as vitamin C, exhibit limited efficacy in mitigating aging-associated oxidative damage because of their lack of mitochondrial targeting and the potential reduction in lifespan associated with high-dose administration. Exercise mimetics can mitigate oxidative damage by targeting and modulating oxidative stress. On the one hand, exercise activates the Nrf2/ARE pathway, boosting the expression of antioxidant enzymes such as superoxide dismutase (SOD) and glutathione peroxidase (GPx), thereby clearing ROS and limiting oxidative damage [192, 193]. On the other hand, exercise mimetics can counteract aging by specifically enhancing mitochondrial homeostasis and biogenesis. Through enhanced autophagy and proteasomal activity, exercise clears damaged proteins and organelles, such as dysfunctional mitochondria, in order to maintain cellular homeostasis [194, 195]. Metformin has been shown to prevent age-dependent hippocampal neuronal loss and reduce oxidative stress [78, 196]. Irisin enhances mitophagy to eliminate damaged mitochondria, thereby reducing oxidative stress and delaying the aging process [197, 198]. Exercise also induces beneficial epigenetic modifications and the clearance of senescent cells. It modulates DNA methylation and microRNA expression, thereby facilitating the transcription of genes involved in neurogenesis and plasticity [194, 199]. Exercise also attenuates cellular senescence by reducing markers such as p16 and p21 and attenuating the deleterious effects of the senescence-associated secretory phenotype (SASP) [200].

Second, with the general extension of lifespan, the incidence of age-related frailty has risen markedly. Reductions in muscle and bone mass not only contribute to chronic pain and loss of mobility, compromising the independence of older adults, but also increase the risk of adverse outcomes and ultimately shorten lifespan [201]. Exercise improves muscle mass and strength, extends lifespan in aged female mice, and enhances bone density and physical function while reducing the risk of falls. Owing to their broad benefits in mitigating aging and promoting healthy longevity, exercise mimetics represent promising strategies for lifespan extension. The exercise mimetic irisin exhibits pronounced skeletal protective effects. Serum irisin levels are positively correlated with bone mineral density [202] and demonstrate significant protective effects across multiple bone disease models and clinical studies [203–205]. Long-term exercise enhances irisin production and secretion in skeletal muscle and upregulates the expression of osteogenic markers [206]. Exogenous irisin mimics the effects of exercise by promoting osteoblast proliferation [207]. Both AICAR and irisin facilitate osteoblast differentiation [206, 208], while PPARδ agonists further augment bone formation in mice. Irisin also stimulates pre-osteoclast proliferation and suppresses osteoclast maturation [209], thus regulating skeletal homeostasis through multiple mechanisms.

Third, neurotrophic support is generally reduced in aging individuals, as characterized by decreased neurotrophic factor expression, impaired signaling pathways, reduced neurogenesis, and increased neuroinflammation, all of which contribute to neuronal vulnerability and cognitive decline [210]. Building on these mechanistic insights, several studies have explored whether mimicking individual neurotrophic signals can reproduce exercise-like benefits in aging models. For instance, the BDNF mimetic 7,8-DHF enhances the dendrite maturation of newborn hippocampal neurons, thereby alleviating age-related structural deficits [211]. Similarly, IGF-1 gene therapy augments hippocampal neurogenesis and astrocyte branching and enhances spatial memory in female aging rats [212]. Moreover, VEGF preconditioning facilitates NSC remodeling and supports structural and functional resilience during aging [213]. In addition, recent studies have identified betaine as a potential exercise mimetic capable of alleviating cellular senescence and exerting broad longevity-promoting effects across multiple organ systems [36, 66].

These findings suggest that exercise mimetics play important roles in delaying aging, mimicking the skeletal protective effects of exercise, maintaining cardiovascular health, reducing mortality risk in older adults, and extending the healthy lifespan.

### Vascular and angiogenic adaptations

Exercise improves cerebral blood flow and the vascular microenvironment, thereby maintaining brain structural integrity (Fig. 3d). Both acute and chronic exercise can enhance cerebral perfusion by upregulating eNOS, ensuring adequate oxygen and nutrient supply, particularly in regions vulnerable to atrophy such as the hippocampus [194]. These vascular benefits are further augmented by increased BBB permeability, which facilitates the delivery of neurotrophic factors and metabolic substrates to the aging brain [214]. Exercise mimetics have also been implicated in the vascular and angiogenic adaptations that contribute to cardiometabolic and cerebrovascular health. Chronic exercise markedly increases circulating irisin levels, which correlate positively with VO₂max and are associated with improved endothelial function and reduced cardiometabolic risk. Elevated irisin can inhibit cardiac hypertrophy and support cardiovascular protection. In addition, NAD⁺ boosters such as nicotinamide mononucleotide (NMN) enhance endothelial angiogenic programs and improve neurovascular coupling, thereby promoting tissue perfusion in a manner analogous to exercise-induced vascular adaptations [70]. Although preclinical evidence supports the promise of NAD⁺ boosters, human clinical trials have shown limited efficacy [215]. Similarly, the AMPK agonist metformin has been shown to stimulate cerebral angiogenesis and improve cognitive performance in experimental models [216].

Beyond its effects on cerebral angiogenesis and vascular regulation, exercise exerts widespread and profound impacts on the systemic vasculature. Exercise directly enhances endothelial function and promotes angiogenic regulation, thereby optimizing blood supply and the tissue microenvironment [217]. Similarly, key exercise-related molecules and their mimetics can partially reproduce the vascular protective effects of physical activity. Irisin targets endothelial cells and promotes angiogenesis through multiple pathways [218]. In vivo and in vitro studies have shown that irisin upregulates the expression of angiogenesis-related genes [219], enhances the migration and tube formation of human umbilical vein endothelial cells [220, 221], and activates the ERK signaling pathway in endothelial cells to promote capillary formation [221]. Moreover, the exercise mimetic trimetazidine also exhibits vascular regulatory activity, increasing endothelial cadherin expression and elevating vascular endothelial growth factor levels in skeletal muscle [171].

In summary, exercise enhances endothelial function and promotes angiogenesis, thereby systematically optimizing blood circulation and the tissue microenvironment. Exercise mimetics exhibit similar vascular regulatory potential, offering new avenues for the integrated prevention and treatment of cardiovascular and cerebrovascular diseases.

### Immune modulation and inflammation

Long-term exercise markedly regulates immune function. Regulatory T cells (Tregs) play a central role in maintaining immune homeostasis and tolerance. Irisin mimics the effects of exercise by interacting with Treg receptors, promoting their proliferation and immunosuppressive activity. It may also alter promoter methylation in Treg-specific genes through epigenetic mechanisms, thereby stabilizing their suppressive function and longevity [222]. In macrophages, irisin normalizes pro-inflammatory cytokine levels, suppresses p53-mediated apoptosis and inflammasome activation, and exerts potent anti-inflammatory effects [223]. These actions create a favorable microenvironment for Treg-mediated immune regulation [224].

Moreover, an imbalance in the inflammatory microenvironment is a major trigger of immune dysfunction. Exercise exerts potent anti-inflammatory and antioxidant effects (Fig. 3e). It reduces microglial overactivation and polarization toward the pro-inflammatory phenotype, decreasing the release of pro-inflammatory cytokines such as IL-1β and TNF-α while enhancing the release of anti-inflammatory mediators such as IL-10, CLU, and C-X3-C motif chemokine ligand 1 (CX3CL1) [34, 225]. Betaine exerts anti-inflammatory effects by inhibiting the TBK1/IRF3/p65 signaling pathway, thereby reducing the expression of pro-inflammatory cytokines such as TNF-α and IL-1β [36]. The exercise mimetic trimetazidine enhances ATP production, mitigates neuroinflammation and oxidative damage, and exhibits strong anti-inflammatory potential [226].

Beyond exerting anti-inflammatory and neuroprotective effects in the central nervous system, exercise-induced molecular responses also mediate broad anti-inflammatory and restorative actions in peripheral tissues. The myokine irisin suppresses macrophage infiltration, activates the Nrf2/HO-1 antioxidant pathway, and promotes macrophage polarization toward the reparative M2 phenotype [227–229]. Simultaneously, the exercise-induced ketone body BHB acts as a key signaling metabolite that modulates the immunometabolic microenvironment by inhibiting the macrophage cGAS-STING pathway and driving M2 polarization [177]. Additionally, the newly identified exercise metabolite N-lactoyl-phenylalanine (Lac-Phe) exhibits tissue-specific action, inhibiting NF-κB signaling to block the pro-inflammatory M1 polarization of intestinal macrophages [230]. Collectively, the synergistic activities of irisin, BHB, and Lac-Phe establish an integrated multi-organ anti-inflammatory network, thereby providing a mechanistic foundation for the development of exercise mimetics.

Collectively, these findings support the view that both exercise and exercise mimetics are potent, multimodal interventions that enhance the body’s regenerative potential. Exercise mimetics reproduce key molecular cascades originally activated by physical activity, including metabolic regulation, neurotrophic signaling, redox homeostasis, organ protection, angiogenesis, and immune modulation. For patients with diseases characterized by related physiological alterations, mimetics provide a promising pharmacological strategy to harness the regenerative benefits of exercise for disease treatment.

## Exercise mimetics in major human diseases

Recent studies have shown that both exercise and exercise mimetics can promote regeneration through multidimensional mechanisms, achieving multi-target synergistic effects that are difficult to attain with single-target drugs [6]. The following sections specifically discuss the effects and mechanisms of exercise and exercise mimetics on regeneration in cancer, traumatic injuries, cardiovascular disorders, neurodegenerative diseases, and immune-mediated diseases (Table 2).

**Table 2:** Potential exercise mimetics for the treatment of major human diseases

Disease	Exercise mimetics	Category	Biological functions	References
Cancer	Irisin	Myokine	Enhances blood–brain barrier permeability and antitumor drug delivery, induces cell cycle arrest and apoptosis, and remodels the tumor microenvironment to suppress tumor progression	[231–234]
Traumatic injuries	TZDs	PPARγ agonist	Reduce neuroinflammation and microglial activation	[235]
	7,8-DHF	BDNF mimetic	Promote survival of neurons	[236]
	R-13	BDNF mimetic	Promote axon regeneration	[48]
	IGF-1	Growth factor	Increase immature granule cells in the hippocampus	[237]
	AICAR	AMPK agonist	Promote mitochondrial biogenesis	[238]
Cerebrovascular disease	NO	Guanylyl cyclase agonist	Improve cerebral perfusion and reduce vascular dysfunction	[239, 240]
	Irisin	Myokine	Enhance neurovascular unit integrity; reduce pro-inflammation cytokines expression and promote microglial polarization toward an anti-inflammatory phenotype	[241–243]
	QK peptide	VEGF mimetic	Promote neovascularization	[244]
	Metformin	AMPK agonist	Suppress microglia overactivation; restore OPC function	[245, 246]
	IL-4 nanoparticles	Cytokine	Promote oligodendrogenesis	[247]
	LM22A-4	BDNF mimetic	Promote neurogenesis	[39, 49]
Alzheimer’s Disease	7,8-DHF	BDNF mimetic	Reduce Aβ accumulation and synapses loss	[248–250]
	Irisin	myokine	Reduce Aβ accumulation, tau phosphorylation and neuroinflammation	[119, 251]
	Pioglitazone	PPARγ agonist	Increase Aβ phagocytosis and suppress IL-1β	[84]
	Metformin	AMPK agonist	Reduce Aβ and tau pathology, and restore hippocampal neurogenesis	[252]
	CLU	Hepatokine	Reduce neuroinflammation gene expression	[34]
	SAM	Methyl donor	Reduce Aβ accumulation, tau hyperphosphorylation, oxidative stress, inflammation, mitochondrial dysfunction	[113, 249]
	miR-132	MicroRNA	Reduce Aβ accumulation and tau hyperphosphorylation,	[103, 104]
Multiple Sclerosis	Metformin	AMPK agonist	Inhibit proinflammation cytokines (IFN-γ, TNF-α, IL-6, IL-17) and increase IL-10; promote OPC differentiation; enhance mitochondrial function and ATP supply	[253–256]
	Rolipram	cAMP/CREB agonist	Suppress the production of TNF-α and LT-α; promote OPC maturation via MEK-ERK pathway	[257, 258]
	Forskolin	cAMP/CREB agonist	Inhibit IL-17-STEAP4 signaling pathway and restrain Th17 differentiation; promote myelin gene transcription	[86, 259]
	DMF	Nrf/ARE activator	Inhibit IL-12/IL-23-driven Th1/Th17 responses and reinforce anti-inflammatory programs	[260]
	Irisin	Myokine	Reduce NF-κB signaling and limiting microglial activation	[261]
	7,8-DHF	BDNF mimetic	Support oligodendrocyte survival and myelin repair	[262]
	MitoQ	Mitochondria-targeted mimetic	Mitigate redox stress and stabilize mitochondrial dynamics	[88]
	Rosiglitazone	PPARγ	Increase blood–brain barrier integrity	[263]

### Cancer biology

Exercise not only reduces the incidence of multiple cancers and delays tumor progression, but also provides adjunctive benefits for cancer treatment and patient recovery [264]. These benefits have also been demonstrated in highly aggressive glioblastoma. Glioblastoma is the most common and highly aggressive grade IV brain tumor, with a median survival of only 10–12 months after diagnosis. Emerging evidence suggests that exercise may provide potential benefits during treatment and recovery, and survival in recurrent malignant glioma correlates positively with physical activity levels [265].

Exercise modulates the epigenetic regulation of gene expression and immune function, restores immune balance, and suppresses tumor initiation, growth, and metastasis [231]. Exercise releases factors such as FNDC5, irisin, and IGF-1, which cross the blood–brain barrier and regulate glioma cells. These factors enhance mitochondrial function, reduce inflammation, and regulate glioma signaling. On the one hand, irisin improves BBB permeability, enhancing the penetration and targeting of antitumor drugs, and thereby offering new strategies for brain tumor therapy [232]. On the other hand, it induces cell cycle arrest and activates apoptotic pathways, thereby inhibiting tumor cell proliferation and promoting programmed cell death [233, 234]. Furthermore, irisin remodels the tumor microenvironment, thereby further suppressing tumor invasion and metastasis. The exercise mimetic irisin shows remarkable potential in the treatment of highly malignant tumors such as glioblastoma. Further research on exercise mimetics could consolidate their role in the future clinical management of glioma.

### Traumatic injuries

**Fig. 4:**
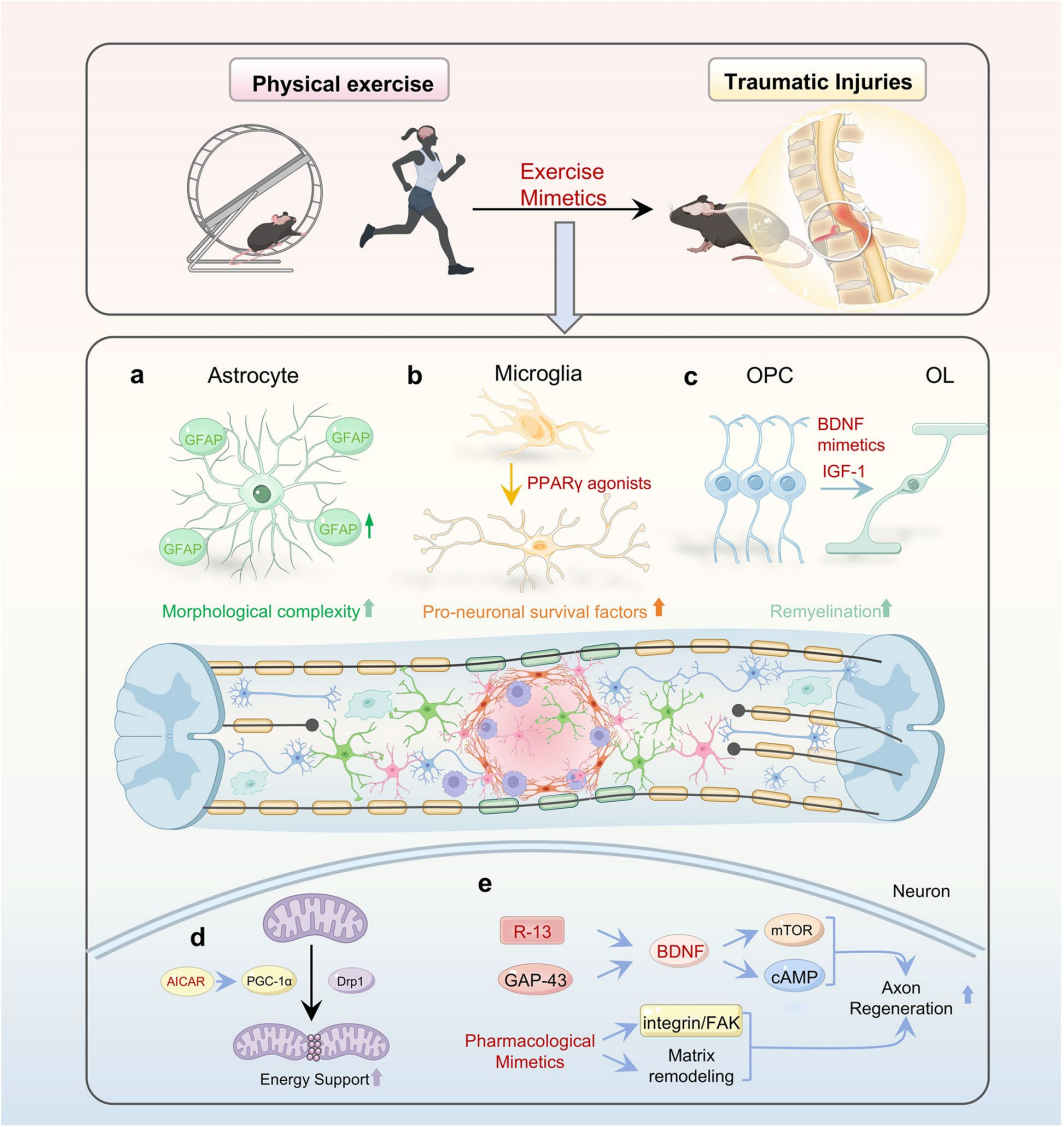
Exercise- and exercise mimetic-induced mechanisms of neuroregeneration after traumatic injury. **a** Exercise and exercise mimetics increase astrocyte density, GFAP expression, and morphological complexity, supporting neuronal repair. **b** PPARγ agonists shift microglia toward pro-neurogenic (e.g., anti-inflammatory) phenotypes, enhancing neuronal survival and vascular protection. **c** BDNF and IGF-1 promote OPC differentiation into oligodendrocytes, facilitating axonal remyelination. **d** AICAR activates PGC-1α signaling, enhancing mitochondrial biogenesis and Drp1-mediated dynamics to sustain energy metabolism. **e** Exercise and exercise mimetics stimulate axonal regeneration and synaptic plasticity through distinct mechanisms: R-13 and GAP-43 promote axon growth, whereas mimetics that enhance BDNF/TrkB signaling or integrin/FAK-mediated extracellular matrix remodeling promote neurogenesis and neuroplasticity

Traumatic injuries to the CNS, particularly spinal cord injury, present significant barriers to neural regeneration owing to a hostile microenvironment and the limited intrinsic neuronal growth capacity [266, 267]. Exercise has been shown to remodel the inhibitory microenvironment, restore mitochondrial function, activate pro-regenerative signaling cascades, and promote endogenous stem cell proliferation (Fig. 4). Importantly, many of these effects can also be reproduced by exercise mimetics that target the corresponding molecular pathways.

First, exercise modifies the inhibitory post-injury microenvironment. It modulates astrocytic responses by increasing glial fibrillary acidic protein (GFAP) expression and morphological complexity [268] (Fig. 4a). Concurrently, exercise attenuates microglial overactivation, shifts microglia toward a pro-neuronal survival phenotype, and enhances their neurotrophic and phagocytic functions to clear inhibitory molecules [269] (Fig. 4b). Similarly, exercise mimetics that modulate microglial activation, such as the PPARγ agonist thiazolidinediones (TZDs), can recapitulate these anti-inflammatory effects, thereby safeguarding neuronal integrity and establishing a more permissive regenerative microenvironment [235]. Moreover, treadmill exercise upregulates the BDNF, IGF-1, and PGC-1α pathways, enhancing oligodendrocyte precursor cell (OPC) maturation and the expression of myelin-associated proteins, ultimately contributing to remyelination and facilitated conduction [270, 271] (Fig. 4c). These changes collectively help reverse the inhibitory microenvironment, facilitating axonal penetration through glial scar regions. These effects of exercise have also been recapitulated by exercise mimetics that target BDNF or IGF-1 signaling. For example, the BDNF mimetics R-13 and 7,8-DHF reduce the apoptosis of cortical neurons and attenuate brain tissue damage after traumatic brain injury [236, 272]. Administration of synthetic IGF-1 increases the number of immature granule cells in the hippocampus and prevents traumatic brain injury-induced gastrointestinal dysfunction [237], suggesting its potential to overcome the inhibitory post-injury microenvironment.

Second, exercise enhances mitochondrial dynamics and energy metabolism, which are critical for the high energy demands of axon regeneration. It promotes mitochondrial biogenesis and transport by upregulating the expression of mitochondrial dynamics-related proteins such as dynamin-related protein 1 (Drp1) and PGC-1α [273] (Fig. 4d). Following knockout of the mitochondrial anchoring protein syntaphilin in a mouse model of spinal cord injury, treadmill training dramatically boosts corticospinal tract axon regeneration across lesion sites [274]. Exercise mimetics such as AICAR (an AMPK agonist) mimic these effects by activating the AMPK-PGC-1α pathway and restoring mitochondrial efficiency [238].

Third, exercise reactivates axonal growth programs through the upregulation of canonical regenerative pathways. It enhances the expression of growth-associated protein-43 (GAP-43), an important marker of axonal regeneration, and increases BDNF levels. Elevated BDNF subsequently activates signaling pathways such as mTOR and the cyclic adenosine monophosphate (cAMP), which promote axon extension and neuronal survival in injured tissue [275–277] (Fig. 4e). BDNF mimetics (e.g., R-13) have been shown to reproduce these effects pharmacologically [48].

Fourth, exercise stimulates the proliferation and lineage specification of NSCs and neural progenitor cells (NPCs) [278]. The mechanical stress generated during physical activity activates integrin-mediated signaling and extracellular matrix remodeling, increasing the expression of molecules such as tenascin that guide neuronal migration and differentiation [20], thereby enhancing neurogenesis and neuroplasticity. Pharmacological mimetics that enhance integrin/FAK signaling or extracellular matrix (ECM) remodeling may provide comparable neurogenic support.

Taken together, these findings underscore that exercise establishes a multifaceted regenerative program in traumatic injuries, acting through glial modulation, mitochondrial reprogramming, axonal growth activation, and neurogenesis. Exercise mimetics that target these same pathways, such as AMPK agonists, PPARγ activators, BDNF mimetics, mitochondrial antioxidants, and ECM-modulating agents, provide a pharmacological means of reproducing the benefits of physical training. By operationalizing the regenerative logic of exercise in drug form, such mimetics hold promise as adjunctive or alternative strategies for patients with spinal cord injury who cannot reliably engage in intensive rehabilitation.

### Cerebrovascular disorders

**Fig. 5:**
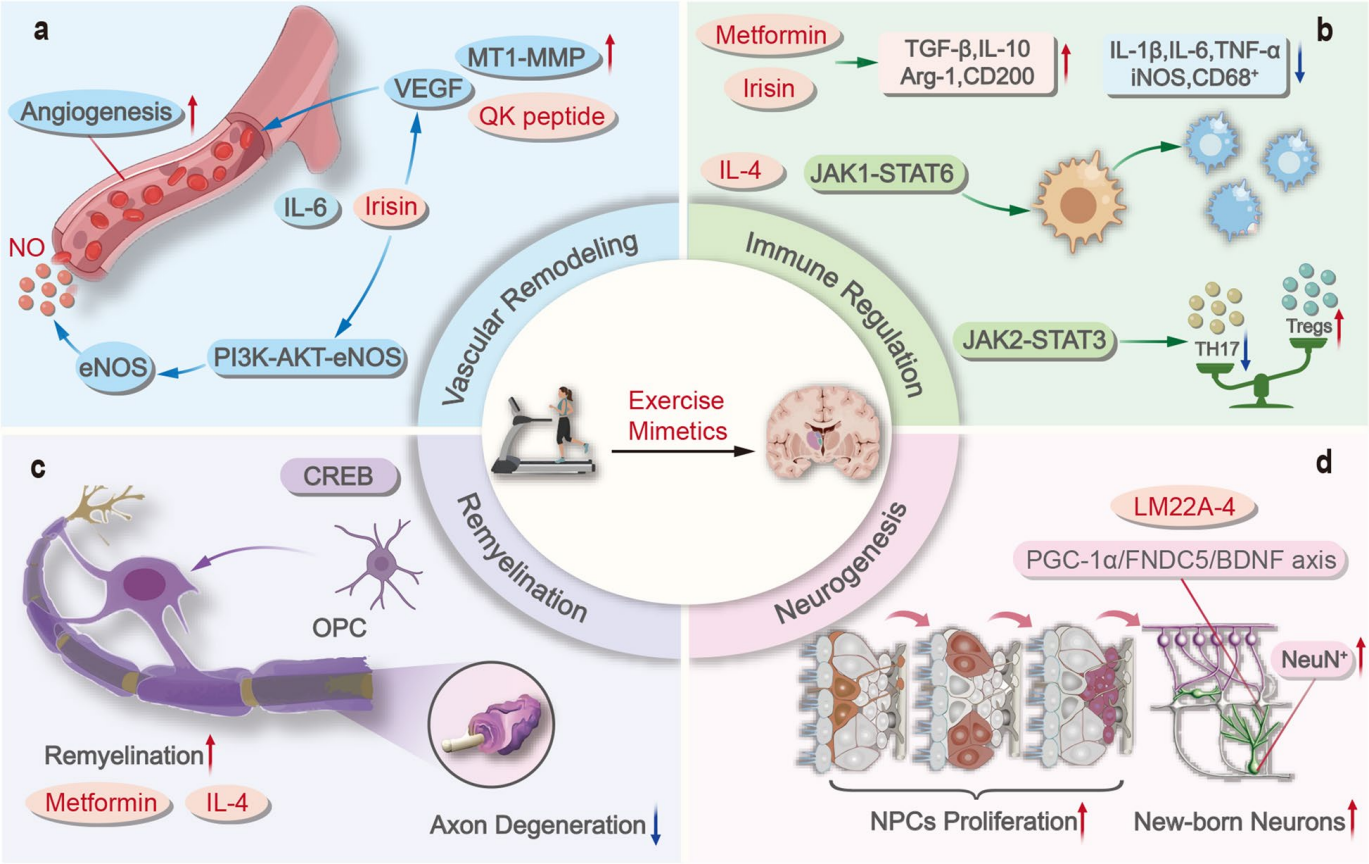
Exercise- and exercise mimetic-induced mechanisms of neuroregeneration in cerebrovascular disorders. **a** Exercise mimetics such as IL-6, irisin, and the QK peptide activate PI3K-AKT-eNOS signaling and upregulate VEGF and MT1-MMP, thereby promoting angiogenesis and vascular remodeling. **b** Exercise mimetics (e.g., metformin, irisin, and IL-4) activate the IL-4-JAK1-STAT6 pathway to induce anti-inflammatory microglial polarization, while suppressing the JAK2-STAT3 axis to restore Th17/Treg balance and reduce neuroinflammation. **c** Exercise mimetics (e.g., metformin and IL-4) stimulate CREB signaling and OPC differentiation, enhancing remyelination and mitigating axonal degeneration. **d** Exercise mimetics (e.g., LM22A-4) activate the PGC-1α/FNDC5/BDNF axis, driving NPC proliferation and the integration of newborn NeuN⁺ neurons, thereby supporting neurogenesis and synaptic plasticity

Exercise promotes neurovascular regeneration and functional recovery following cerebrovascular injury through angiogenesis, immune modulation, remyelination, and neurogenesis (Fig. 5). Exercise mimetics targeting vascular and immune pathways have demonstrated similar effects.

First, exercise promotes angiogenesis and vascular remodeling (Fig. 5a). In the pathological context of myocardial infarction, exercise activates key signaling cascades, such as the phosphatidylinositol 3-kinase/protein kinase B (PI3K/Akt) pathway, leading to eNOS phosphorylation and increased nitric oxide production [279]. This vasodilatory mediator improves cerebral perfusion and reduces vascular inflammation, thereby preserving the integrity of the neurovascular unit [280]. Moreover, exercise stimulates the release of angiogenic myokines via the muscle-brain axis, including IL-6, irisin, and follistatin-like 1 (FSTL1), upregulating VEGF and membrane-type I matrix metalloproteinase (MT1-MMP) to facilitate endothelial cell migration and lumen formation [281]. VEGF serves as a bridge between vascular and neurogenic repair. Peripheral inhibition of VEGF signaling has been shown to abrogate exercise-induced hippocampal neurogenesis, highlighting its dual role in angiogenesis and neural plasticity [282]. Building on these findings, exercise mimetics that selectively recapitulate cardiovascular responses to physical activity may confer comparable vasoprotective benefits [31]. For instance, exogenous NO supplementation, delivered through classical donors, has been shown to preserve endothelial function during ischemia–reperfusion, improve cerebral perfusion, and reduce vascular dysfunction [239, 240]. Recombinant irisin reduces infarct size, blood–brain barrier permeability, and neuronal apoptosis in rodent models of middle cerebral artery occlusion, while upregulating BDNF and enhancing neurovascular unit integrity [241]. Complementarily, the VEGF-mimetic QK peptide, a synthetic helix derived from the VEGF receptor-binding domain, activates angiogenic signaling and promotes neovascularization in vivo, thereby representing a more stable and controllable VEGF mimetic with translational potential in post-stroke vascular remodeling [244]. These agents highlight how vascularly oriented exercise mimetics can restore perfusion, stabilize the blood–brain barrier, and stimulate angiogenesis, offering a mechanistically grounded strategy to promote cerebrovascular repair when physical exercise is limited.

Second, exercise reshapes immune-neural interactions within the injured brain (Fig. 5b). In a rat middle cerebral artery occlusion model, treadmill training promotes the polarization of microglia toward an anti-inflammatory phenotype by activating the Janus kinase 1-signal transducer and activator of transcription 6 (JAK1-STAT6) pathway and upregulating IL-4 expression [18]. This results in elevated mRNA levels of repair-associated genes such as IL-10, TGF-β, arginase-1 (Arg-1), and cluster of differentiation 200 (CD200), along with a decrease in pro-inflammatory mediators such as IL-1β, IL-6, TNF-α, inducible nitric oxide synthase (iNOS), and CD68 [18, 283]. Concurrently, exercise inhibits the JAK2/STAT3 axis to restore the T helper 17 (Th17)/Treg balance, although excessive Treg activation may warrant precise regulation in clinical scenarios [19, 284]. Pharmacological exercise mimetics are being explored for their capacity to modulate immune-neural interactions in cerebrovascular disease. For example, AMPK activators such as metformin suppress microglial overactivation and restore anti-inflammatory signaling in the middle cerebral artery occlusion (MCAO) models [245]. Similarly, recombinant irisin administration reduces the expression of pro-inflammatory cytokines such as TNF-α and IL-6, while increasing BDNF, reflecting a neuroprotective and immunomodulatory profile [242]. Furthermore, irisin has been shown in vitro to promote microglial polarization toward an anti-inflammatory phenotype by increasing Arg-1 expression and downregulating NLRP3 inflammasome components [243]. These agents recapitulate key immune-neural adjustments induced by exercise, offering mechanistic support for exercise mimetic strategies in post-stroke repair.

Third, exercise also contributes significantly to myelin regeneration and white matter repair (Fig. 5c), a key determinant of cognitive and motor function in cerebrovascular diseases. Aerobic activity improves microvascular perfusion in subcortical white matter regions and enhances OPC proliferation and differentiation [285]. Combinatorial therapies, such as electroacupuncture combined with treadmill training, amplify these effects through activation of the cAMP response element-binding protein (CREB)/BDNF pathway, restoring myelin integrity after hypoxic-ischemic injury and improving behavioral outcomes [285]. Several exercise-mimetic strategies foster remyelination after cerebrovascular injury by converging on exercise-responsive pathways. For example, intranasal delivery of IL-4 nanoparticles, a mimetic for the exercise-induced IL-4/PPARγ axis, robustly promotes oligodendrogenesis, enhances white-matter integrity, and improves long-term function after experimental stroke, establishing a direct causal link between IL-4 signaling and post-ischemic remyelination [247]. In addition, the AMPK activator metformin alleviates white-matter damage and restores OPC function in a chronic cerebral hypoperfusion model [246], further supporting AMPK-driven remyelination in cerebrovascular disease.

Fourth, exercise promotes adult neurogenesis and synaptic remodeling (Fig. 5d). Treadmill training increases hippocampal neurogenesis and enhances memory and motor recovery after stroke, at least partly by upregulating synaptic proteins such as postsynaptic density protein 95 and synaptophysin [286, 287]. These structural changes are underpinned by activation of the PGC-1α/fibronectin type III domain-containing protein 5 (FNDC5)/BDNF axis, with prolonged endurance training boosting BDNF synthesis across multiple brain regions [29]. Exercise mimetics can engage neurogenic and synaptic programs after ischemic stroke. The small-molecule TrkB agonist LM22A-4, a BDNF mimetic, promoted post-stroke neurogenesis and improved functional recovery when administered after hypoxic-ischemic stroke in mice [49], and LM22A-4-loaded smart mesoporous balls further enhanced cognitive and motor outcomes in ischemic models, consistent with BDNF-driven synaptic plasticity [39].

Taken together, converging evidence indicates that exercise mimetics targeting vascular, immune, myelin, and neurogenic pathways can reproduce key benefits of physical training after cerebrovascular injury, offering a feasible therapeutic avenue for patients who are unable to engage in intensive exercise.

### Neurodegenerative disorders

Neurodegenerative diseases, such as AD, are characterized by progressive neuronal loss and neuroinflammation. Mounting evidence suggests that exercise exerts disease-modifying effects through multifaceted mechanisms, including the enhancement of neurotrophic support, modulation of neuroinflammation, improvement of metabolic homeostasis, and restoration of BBB function.

**Fig. 6:**
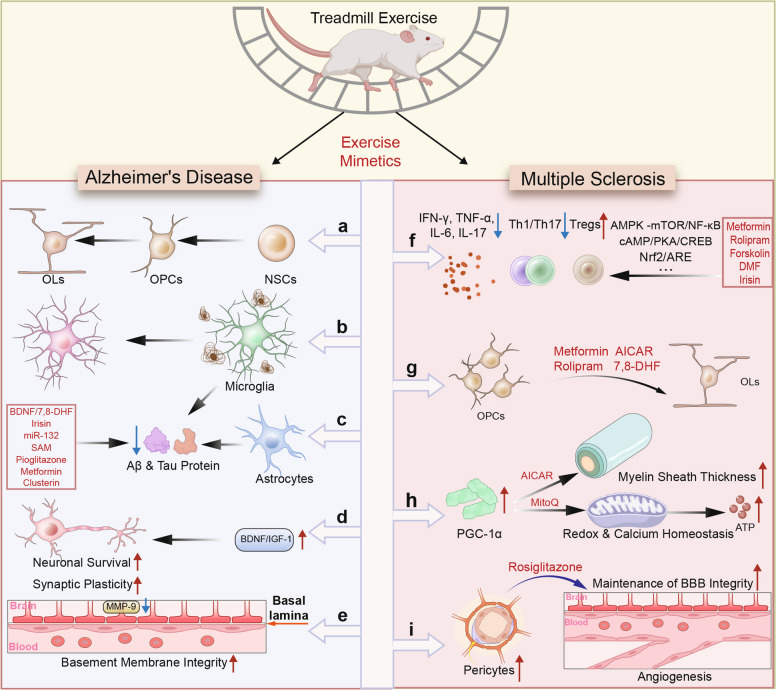
Exercise- and exercise mimetic-induced mechanisms of neuroregeneration in Alzheimer’s disease and multiple sclerosis. **a-e** Alzheimer’s disease (AD). **a** Exercise promotes NSC and OPC proliferation and differentiation, facilitating remyelination and repair. **b-c** Exercise reduces microglial activation and enhances astrocytic AQP4 polarization and neprilysin secretion, thereby facilitating the clearance of Aβ and tau and reducing neuroinflammation. **d** Exercise elevates neurotrophic factors (e.g., BDNF and IGF-1), supporting neuronal survival and synapse integrity. **e** Exercise protects the BBB and basement membrane by suppressing MMP-9. Mimetics (e.g., BDNF/7,8-DHF, irisin, miR-132, SAM, pioglitazone, metformin, and clusterin) reproduce these effects. **f-i** Multiple sclerosis (MS). **f** Exercise reduces CNS infiltration of pro-inflammatory cells and cytokines (IFN-γ, IL-17, and IL-1β), while increasing the number of regulatory T cells. **g** Exercise enhances OPC differentiation into OLs, promoting remyelination. **h** Exercise-driven PGC-1α activation improves mitochondrial bioenergetics and redox and calcium balance, boosting ATP and myelin thickness. **i** Exercise maintains BBB integrity and promotes angiogenesis via pericyte recruitment. Mimetics (e.g., metformin, rolipram, AICAR, forskolin, DMF, irisin, MitoQ, rosiglitazone, and 7,8-DHF) target similar pathways

AD is characterized by β-amyloid (Aβ) accumulation and tau pathology [288]. Accumulating evidence suggests that exercise ameliorates AD-related neuropathology through the enhancement of neurogenesis, glial modulation, myelin regeneration, and BBB protection (Fig. 6a-e).

In amyloid precursor protein/presenilin-1 (APP/PS1) transgenic mouse models, voluntary running promotes the differentiation of OPCs into mature oligodendrocytes (OLs) in the medial prefrontal cortex, leading to remyelination around Aβ plaques [289] (Fig. 6a). This is associated with reduced Aβ deposition and improved learning and memory [289]. Exercise further reshapes glial phenotypes in the AD brain. It shifts microglia toward pro-neural survival states and promotes aquaporin-4 (AQP4) polarization in astrocytes, facilitating the clearance of Aβ and tau via the glymphatic and circulatory systems [290, 291] (Fig. 6b-c). Additionally, exercise-induced irisin activates astrocytes to secrete neprilysin, a key Aβ-degrading enzyme, directly reducing pathological Aβ deposition [8]. Exercise also restores astrocytic vessel coverage in the cortex and hippocampus, which is involved in neurovascular coupling and supports neuronal function [292]. Furthermore, exercise restores the number of newborn neurons in AD mice, reversing the approximately 40% reduction observed in disease states. Molecular profiling has shown that exercise upregulates genes involved in ATP production and lysosomal regulation in immature neurons, contributing to neuronal resilience [293]. Key mediators of these effects include BDNF and IGF-1, both of which are elevated in response to exercise and promote neuronal survival, axonal growth, and synaptic plasticity [46] (Fig. 6d). Preclinical studies also suggest that combining exercise with music therapy improves neuropsychiatric outcomes in patients with mild-to-moderate AD [294]. From a vascular perspective, exercise protects the BBB and basement membrane integrity by downregulating matrix metalloproteinase-9 (MMP-9) and upregulating tissue inhibitors of metalloproteinases (TIMPs) [295] (Fig. 6e). These studies support the role of physical activity in delaying AD progression and preserving cognitive function via BBB stabilization.

The neuroprotective mechanisms of exercise in AD can be pharmacologically recapitulated through a growing class of exercise mimetics. For instance, pharmacological delivery of BDNF or 7,8-DHF markedly reduces Aβ accumulation, inhibits the loss of hippocampal synapses, and restores synaptic plasticity and cognitive performance [248–250]. Recombinant irisin administration attenuates tau phosphorylation and neuroinflammation [251], while also protecting against Aβ-associated deficits [119]. PPARγ agonists such as pioglitazone increase the phagocytosis of Aβ and suppress the pro-inflammatory cytokine IL-1β [84]. Metformin, through activation of the AMPK/PGC-1α cascade, reduces Aβ and tau pathology, restores hippocampal neurogenesis, and improves recognition memory, thereby capturing the metabolic and neurogenic dimensions of exercise [252]. Intravenous injection of clusterin reduces neuroinflammation gene expression in a mouse model of AD [34]. Nevertheless, research has cautioned that chronic treatment with metformin led to impairments in memory retention and discrimination learning at older ages and in AD mouse models [296], casting a shadow over its potential application in the treatment of AD. Supplementation with S-adenosylmethionine (SAM), a methyl donor, decreases hippocampal Aβ deposition and tau hyperphosphorylation, while mitigating oxidative stress, inflammation, mitochondrial dysfunction, and cholinergic deficits [113, 249]. Beyond these protein- or metabolite-based approaches, nucleic acid mimetics have also shown promise. Delivery of synthetic miR-132 restores hippocampal neurogenesis and cognitive function by reducing Aβ burden and tau hyperphosphorylation, whereas intracerebroventricular administration of anti-miR-132 oligonucleotide abolishes the exercise-induced benefits on neurogenesis, cognition, and BDNF expression [103, 104].

Taken together, these findings underscore the therapeutic potential of targeting exercise-related molecular pathways, including BDNF, irisin, pioglitazone, metformin, SAM, and miR-132, as promising strategies for AD intervention.

### Inflammatory and immune-mediated diseases

Multiple sclerosis (MS) is an autoimmune demyelinating disorder characterized by neuroinflammation and myelin loss. Exercise has been shown to modulate both immune responses and myelin dynamics in animal models such as experimental autoimmune encephalomyelitis (EAE) (Fig. 6f-i). Below, we map canonical exercise-responsive mechanisms in MS to mimetic interventions that act on the same pathways.

First, exercise reduces CNS levels of pro-inflammatory cytokines such as interferon-gamma (IFN-γ), IL-17, and IL-1β, and decreases the infiltration of B and T lymphocytes in EAE mice, while increasing CD4⁺CD25⁺ Treg cells in peripheral lymphoid tissues to promote immune tolerance [297–299] (Fig. 6f). Notably, the anti-inflammatory effects are intensity-dependent, with high-intensity swimming suppressing Th1/Th17 responses more effectively than moderate exercise [300]. Agents that phenocopy exercise-evoked immune reprogramming can recapitulate these benefits in MS models. Metformin, through AMPK activation, ameliorates clinical severity in experimental autoimmune encephalomyelitis (EAE) by suppressing the expression of pro-inflammatory cytokines (IFN-γ, TNF-α, IL-6, and IL-17) while enhancing IL-10, thereby shifting the immune balance toward tolerance [253, 254]. Modulators of the JAK/STAT axis and cAMP/CREB activators (e.g., rolipram and forskolin) similarly attenuate pathogenic cytokine signaling and support Treg responses, aligning with the immunological signature of exercise in EAE [257, 259]. Specifically, rolipram treatment suppresses the production of TNF-α and lymphotoxin (LT-α) in the EAE model [257], whereas forskolin treatment inhibits the IL-17-STEAP4 signaling pathway, restrains Th17 differentiation, and limits lesion-associated inflammation [259]. Dimethyl fumarate (DMF), an FDA-approved therapy for relapsing MS, activates the Nrf2/ARE pathway, simultaneously dampens IL-12/IL-23-driven Th1/Th17 responses, and reinforces anti-inflammatory programs in both preclinical and patient studies [260]. Finally, irisin attenuates neuroinflammation and disease progression in EAE by reducing NF-κB signaling and limiting microglial activation, thereby alleviating both clinical and pathological manifestations [261].

Second, exercise promotes OPC activation and myelin regeneration and reduces axonal damage (Fig. 6g). In demyelinating environments, such as those induced by lysophosphatidylcholine injection, exercise significantly increases OPC proliferation and differentiation by activating the PGC-1α pathway, leading to thickened myelin sheaths and an increased number of remyelinated axons [301, 302]. Complementary evidence from cuprizone-treated mice has shown that exercise upregulates myelin-associated proteins such as myelin basic protein (MBP) and 2’,3’-cyclic nucleotide 3’-phosphodiesterase (CNP) [303]. Notably, exercise-induced myelin regeneration is pathology-specific, requiring a demyelinating context to trigger new myelin formation by both newly generated and surviving oligodendrocytes [304, 305]. Additionally, optogenetic stimulation of cortical circuits synergizes with exercise to enhance OPC differentiation at demyelinated sites [305]. Collectively, exercise attenuates neuroinflammation and directly targets oligodendrocyte lineage cells to promote myelin repair and reduce axonal pathology. The AMPK agonist metformin reproduces the pro-myelinating effects of training by increasing OPC differentiation and the repopulation of mature oligodendrocytes in the cuprizone murine model of multiple sclerosis [255]. Forskolin, a cAMP/CREB agonist, promotes myelin gene transcription [86], while rolipram promotes OPC maturation via the MEK-ERK signaling pathway [258], both mirroring exercise-induced remyelination. Neurotrophin mimetics, including BDNF analogues (e.g., 7,8-DHF), further support oligodendrocyte survival and myelin repair, aligning with exercise-upregulated neurotrophic signaling [262]. These data provide a mechanistic basis for exercise-mimetic approaches that target oligodendrocyte lineage cells to rebuild myelin and limit axonal injury in MS.

Third, exercise enhances mitochondrial function and metabolism. Axonal degeneration in multiple sclerosis is strongly associated with impaired mitochondrial energy metabolism [306]. ATP deficits emerge early during acute neuroinflammatory lesions and persist throughout the chronic disease course, reflecting a primary bioenergetic failure that precedes alterations in mitochondrial redox balance and calcium homeostasis [306]. Exercise has been shown to restore metabolic capacity by enhancing mitochondrial biogenesis through activation of the PGC-1α signaling pathway, thereby improving axonal ATP availability and supporting neuroenergetic demands [306] (Fig. 6h). AMPK activators (e.g., AICAR and metformin) recapitulate the mitochondrial program of exercise, augmenting oxidative phosphorylation and axonal ATP supply [256]. The mitochondria-targeted mimetic MitoQ mitigates redox stress, stabilizes mitochondrial dynamics, and alleviates pathogenesis in the EAE mouse model of MS [88]. Together, these agents phenocopy the metabolic reset that underpins exercise-supported remyelination and axonal preservation.

Fourth, exercise contributes to the maintenance of BBB integrity by enhancing the expression of tight junction proteins and increasing pericyte coverage of microvessels, thereby limiting immune infiltration and creating a microenvironment conducive to neural repair [307, 308] (Fig. 6i). The PPARγ agonist rosiglitazone can reproduce exercise-evoked endothelial stabilization and BBB integrity, decreasing the levels of inflammatory mediators and oxidative endothelial injury [263].

These observations support a pathway-guided approach in which exercise-responsive mechanisms in MS are leveraged pharmacologically. These exercise mimetic strategies, including AMPK activators, cAMP/CREB agonists, BDNF mimetics, irisin, DMF, MitoQ, and PPARγ agonists, chart a feasible route to translate the regenerative logic of exercise into therapeutic interventions for MS patients with physical limitations.

Collectively, accumulating evidence indicates that exercise mimetics engage conserved adaptive programs and disease-specific regulatory pathways. In CNS disorders, including traumatic injury, ischemic stroke, Alzheimer’s disease, and multiple sclerosis, common mechanisms specifically involve activating core signaling pathways (BDNF-TrkB, IGF1-AKT-mTOR, AMPK-PGC1α, and Nrf2-ARE) and providing fundamental support such as neuroprotection, promotion of neuroplasticity, improved cellular energy metabolism, and enhanced antioxidant defense. However, precise repair of specific neuropathological states requires refined regulation of disease-specific pathways. In traumatic nerve injury, this involves breaking through physical barriers and promoting axonal regeneration via integrin-FAK-RhoA/ROCK mechanotransduction pathways and Piezo1 channels. In cerebrovascular diseases, it focuses on rescuing the ischemic penumbra, driving angiogenesis and vascular remodeling via VEGF-dependent signaling, and precisely modulating post-stroke inflammation in a spatiotemporal manner to mitigate secondary neural damage. In neurodegenerative diseases, key interventions include clearing abnormal protein aggregates (e.g., via enhanced autophagy), supporting vulnerable neuron survival via neurotrophic signals such as BDNF, and intervening early in neural network dysfunction to slow progression. Thus, an optimal therapeutic strategy should consolidate common signaling pathway support while achieving spatiotemporally precise, synergistic regulation of disease-specific pathways based on the core pathological initiators and progression characteristics of different neurological disorders.

## Clinical and translational application of exercise mimetics

Despite compelling preclinical evidence, translating exercise mimetics from experimental systems into clinical practice remains challenging. Key barriers include uncertainty regarding optimal dosing and exposure duration, blood–brain barrier penetration, long-term safety, and patient heterogeneity. Consequently, successful translation will likely depend not only on pharmacological potency, but also on precision patient selection, validated biomarkers of target engagement, and rational integration with complementary therapeutic strategies. Below, we summarize the current translational progress of exercise mimetics across four interrelated dimensions, highlighting both achievements and remaining gaps.

### Biomarker-guided diagnosis and personalized treatment

Exercise mimetics encompass a diverse array of agents that target metabolic, inflammatory, and stress-response pathways activated by physical activity. However, one of the most salient challenges in clinical translation is the heterogeneity of patient response. Not all patients respond uniformly to mimetic agents. This variability underscores the need for biomarker-guided stratification in both clinical development and therapeutic application.

In the context of exercise mimetics, metabolic biomarkers such as insulin sensitivity indices, glucose utilization rates, and serum lipid profiles have been used not only to define baseline metabolic health, but also to monitor acute molecular responses to agents such as metformin and AMPK activators. For example, improvements in insulin sensitivity and shifts in skeletal muscle transcriptomes have been observed in older adults treated with metformin, reflecting changes consistent with exercise-induced metabolic remodeling, and these signatures have been proposed as mimetic markers of pathway activation [309].

Beyond classical metabolic markers, molecular and circulating biomarkers are gaining traction. Circulating microRNAs (c-miRNAs), which change in response to exercise and regulate pathways related to mitochondrial biogenesis and muscle metabolism, are being investigated as potential predictors of individual responsiveness to both physical training and pharmacological mimetics [310]. Early evidence suggests that distinct c-miRNA profiles may help tailor interventions to maximize benefits while minimizing unnecessary exposure [310].

Furthermore, DNA methylation (DNAm)-based epigenetic clocks are particularly attractive as integrative readouts that compress multi-pathway biology into tractable metrics [311–313]. Exercise has been shown to partially reverse age-associated epigenetic drift, notably in pathways related to neurodevelopment, inflammation, and metabolism [314]. The temporal structure of epigenetic adaptation further argues for longitudinal, minimally invasive monitoring rather than static baseline classification. Phase-specific methylation changes observed across weeks of endurance training suggest that continuous liquid biopsy approaches (e.g., cfDNA methylation profiling) and wearable physiological monitoring (e.g., heart rate variability and cortisol rhythms) could support adaptive dose tuning [315–317]. Algorithms based on reinforcement learning may further refine intervention intensity and duration on the basis of patient-specific feedback [317].

Taken together, a biomarker-rich personalized strategy can enhance the interpretability and predictive power of early-phase trials. Such an approach not only improves the likelihood of detecting meaningful biological effects but also supports precision enrichment in larger efficacy studies, thereby aligning the pharmacological action of exercise mimetics with the underlying biology of individual patients.

### Circulating mediators and drug delivery

While biomarkers help define who may benefit, effective translation also depends on how exercise-like signals are transmitted systemically and delivered to the brain. A growing body of evidence indicates that circulating factors can act as systemic exercise mimetics, coordinating immune, vascular, and neural compartments to promote regeneration. Platelet factor 4 (PF4/CXCL4) exemplifies this concept. Independent studies have shown that PF4 is sufficient to rejuvenate aged hippocampal function by reprogramming immune signaling and reducing neuroinflammation. Systemic PF4 attenuates age-related inflammatory signatures and rescues cognition in aged mice [318], whereas klotho elevates platelet factors, including PF4, to enhance cognition across age groups [319]. Furthermore, exercise-induced platelet release of PF4 restores hippocampal neurogenesis and memory [35].

Beyond platelet-derived factors, unbiased plasma proteomics has accelerated the discovery of systemic mediators linking peripheral organs to brain rejuvenation. A seminal study published in *Science* demonstrated that plasma from exercised aged mice transfers enhanced adult hippocampal neurogenesis and cognition to sedentary aged recipients, identifying the liver enzyme GPLD1 as a key mediator whose systemic elevation recapitulates these benefits without entering the brain [32]. Betaine, a natural methyl donor and osmolyte, exerts neuroprotective and cognition-supportive effects through one-carbon metabolism and antioxidative pathways, representing a safe adjunctive candidate for neuroregeneration [320]. In parallel, clusterin, a complement inhibitor elevated by exercise, mediates the transfer of plasma-induced benefits to the aged brain by dampening neuroinflammation and improving cognition [34]. Together, these findings underscore a shift from single intracellular targets toward systemic, multi-organ signaling axes as tractable exercise mimetics.

A significant area of ongoing research focuses on the development of novel exogenous delivery methods for macromolecule transport, including intranasal routes and nanocapsule technologies, with the aim of overcoming pharmacokinetic barriers [46]. Intranasal delivery has emerged as a promising route for enhancing brain exposure while minimizing systemic burden. For example, metformin encapsulated in a MOF-74-Mg metal–organic framework demonstrates efficient intranasal delivery to the hippocampus and striatum, markedly increasing brain accumulation in mice [37]. This approach opens new possibilities for the targeted brain delivery of exercise mimetics.

Nanocarrier platforms further enable the targeted delivery of macromolecules. Mesoporous silica nanoparticles (MSNs), particularly smart mesoporous spheres (SMB-3), exhibit several distinct advantages over existing delivery systems [321]. Compared with free drugs, MSNs display superior biocompatibility, stability, solubility, and therapeutic efficacy, making them ideal candidates for various biomedical applications. SMB-3 has been used to deliver the BDNF mimetic LM22A-4 selectively to ischemic brain regions, significantly improving neurological, motor, and cognitive function following ischemic stroke, reducing apoptosis and glial cell activation, increasing TrkB and Akt phosphorylation, and promoting neurogenesis. Ultimately, this approach reduces post-stroke brain atrophy compared with LM22A-4 alone [39].

Furthermore, lipid nanoparticles (LNPs) efficiently encapsulate and protect mRNA, facilitating its transfection and intracellular delivery, and thereby offering future opportunities to deliver exercise-induced factors or gene-encoded mimetics across the BBB [38].

### Combination therapy of exercise mimetics and other approaches

Given the broad, multisystem effects of exercise, it is increasingly apparent that single-agent exercise mimetics may be insufficient to reproduce its full therapeutic spectrum. This realization has prompted growing interest in combination strategies that integrate exercise mimetics with complementary pharmacological or lifestyle interventions.

The most conceptually grounded and physiologically interpretable combination strategy pairs pharmacological exercise mimetics with actual endurance training. Such studies directly address whether mimetics can amplify training-induced adaptations or partially substitute for exercise when physical activity is limited. For example, in a mouse model, the PPARδ agonist GW501516 induced exercise-like transcriptional programs in sedentary animals and produced additive elevations in serum unsaturated fatty acid levels when combined with endurance running, resulting in superior running performance compared with either intervention alone [322]. However, undesirable side effects and health concerns have precluded the use of GW501516 by athletes for performance enhancement [323].

Pharmacological combinations inspired by exercise-responsive pathways have also been explored. For example, AMPK-activating mimetics (such as AICAR or metformin) have been experimentally combined with mTOR inhibitors (e.g., rapamycin) to reduce kidney tumor size in a nude mouse model [324].

At present, systematic investigations of exercise mimetics in combination with other therapeutic modalities remain relatively limited. This gap represents an important opportunity for future studies to define rational combination strategies that more fully capture the multisystem benefits of physical activity.

### Clinical trials and efficacy

Although exercise mimetics have shown substantial promise in preclinical studies, their translational value for neuroregeneration ultimately depends on clinical evidence. To date, clinical development in this field remains at an early stage, and the available efficacy data are limited and inconsistent across compounds, indications, and trial designs. A balanced evaluation therefore requires not only an examination of registered clinical trials but also consideration of the investment landscape driving development and the regulatory progress achieved so far. These key aspects are summarized in Tables 3 and 4.

**Table 3:** Registered clinical trials of exercise mimetics in neuroregeneration

Drug/Agent	Indication	Phase/Design	NCT Identifier(s)	Status
Metformin	Alzheimer’s disease/MCI	Phase II-III, RCTs (MAP, pilot studies)	NCT04098666; NCT01965756; NCT00620191; NCT02432287	Ongoing/completed
	Frailty/aging	Pilot, RCT	NCT03451006	Terminated (recruitment)
Rosiglitazone	Alzheimer’s disease	Phase II-III (monotherapy and adjunct)	NCT00428090; NCT00381238; NCT00550420; NCT00490568; NCT00348309; NCT00348140; NCT00265148	Completed (negative/inconclusive)
Pioglitazone	AD prevention (TOMMORROW)	Phase III, RCT	NCT01931566	Terminated (futility)
T3D-959	Mild-moderate AD	Phase IIa, proof-of-concept	NCT02560753	Completed (early signals)
Dimethyl fumarate (DMF)	Multiple sclerosis (RRMS)	Phase III (DEFINE, CONFIRM); Phase IV tolerability	NCT00420212; NCT00451451; NCT01873417	FDA approved (2013, RRMS)

**Table 4:** Funding landscape of representative clinical trials

Drug/Agent	Trial (NCT)	Major funding source(s)
Metformin	NCT04098666 (MAP)	NIH/NIA (U01AG058635); multicenter U.S. academic funding
Metformin	NCT00620191	NIH/NIA R01AG026413; Alzheimer’s Drug Discovery Foundation (ADDF #270,901)
Metformin (frailty/aging)	NCT03451006	Mayo Clinic; U.S. institutional support; aging research consortia
T3D-959	NCT02560753 (PIONEER)	NIH/NIA; T3D Therapeutics (industry–academic collaboration)
Pioglitazone	NCT01931566 (TOMMORROW)	Industry-sponsored (Takeda, Zinfandel); biomarker risk algorithm consortia
Dimethyl fumarate (DMF)	NCT00420212/NCT00451451	Biogen (DEFINE, CONFIRM pivotal trials)

Early translational efforts have focused on agents that recapitulate the metabolic and anti-inflammatory pathways linked to exercise, with metformin receiving the most sustained clinical evaluation in AD and mild cognitive impairment (MCI). Multiple trials have examined cognitive outcomes, neuroimaging, and fluid biomarkers in at-risk or prodromal populations (NCT04098666, NCT01965756, NCT00620191, NCT02432287, and NCT03451006). Among these, the multicenter MAP study (NCT04098666) is particularly notable for its scope and methodological rigor. Enrolling approximately 326 nondiabetic adults with amnestic MCI, this trial tests metformin XR (titrated to 2,000 mg/day) over an 18-month period, with a prespecified primary endpoint of verbal memory (FCSRT Total Recall) and a comprehensive secondary battery encompassing global cognition (ADCS-PACC), structural MRI, white-matter hyperintensity burden, amyloid/tau PET, and plasma biomarkers. Enrollment is complete and follow-up is ongoing, with results anticipated to inform whether this low-cost metabolic modulator can meaningfully slow early AD-related decline.

Smaller studies have yielded mixed findings. A randomized cross-over trial (NCT01965756) reported no significant effects on ADAS-Cog word-list memory or CSF Aβ/tau levels over 16 weeks, despite acceptable safety (including transient lactate elevations in 10% of participants without serious events). Earlier placebo-controlled work in aMCI (NCT00620191) suggested selective gains in verbal learning (SRT total recall), but without concordant improvements in global cognition or PET/biomarker readouts over 12 months, highlighting a pattern of domain-specific effects that have not yet translated into robust clinical benefit. Beyond cognition, short-duration mechanistic studies in older adults (NCT02432287) demonstrated that metformin can remodel skeletal muscle and adipose transcriptomes and improve insulin sensitivity, findings consistent with an exercise-like metabolic signature. However, the direct relevance of these findings to neuroregenerative endpoints has yet to be established. A small frailty pilot study (NCT03451006) further indicated functional gains on the Short Physical Performance Battery (SPPB) and improvements in inflammatory markers, although the limited sample size precludes definitive interpretation.

These mixed outcomes have spurred a critical reassessment of other metabolic strategies inspired by exercise-responsive signaling. In this context, PPAR-γ-directed approaches have produced largely negative or equivocal results in Alzheimer’s disease. Across several phase II/III programs of rosiglitazone XR, including adjunctive and monotherapy designs with APOE-ε4 stratification (e.g., NCT00428090, NCT00348309, and NCT00348140) and extended open-label safety cohorts (NCT00381238, NCT00490568, NCT00550420, and NCT00265148), no reproducible treatment advantage emerged on the ADAS-Cog, CDR-SB, or global clinical impressions relative to standard-of-care and placebo. Signals in ε4-negative subgroups were inconsistent and failed to cross prespecified efficacy thresholds, while safety generally aligned with known class effects (e.g., edema). Similarly, the TOMMORROW trial (pioglitazone 0.8 mg/day; NCT01931566) validated a biomarker-based risk algorithm but did not demonstrate a reduction in the risk of MCI-AD conversion versus placebo, leading to early termination for futility. Collectively, these data suggest that broad PPAR-γ agonism is unlikely to confer clinically meaningful disease modification in unselected AD populations, and that future success will require more precise pharmacology or more rigorous enrichment strategies.

By contrast, next-generation metabolic rewiring approaches are beginning to show promising target engagement with favorable pharmacokinetics. T3D-959, a dual PPAR-δ/γ modulator designed to normalize cerebral glucose utilization, produced dose-dependent changes in fluorodeoxyglucose positron emission tomography (FDG-PET)-measured metabolism and hippocampal functional connectivity over 14 days in mild-to-moderate AD (NCT02560753), with acceptable short-term tolerability. Although cognitive outcomes (ADAS-Cog11 and DSST) did not achieve statistical significance within this brief treatment window, the imaging pharmacodynamics provided evidence of CNS penetration and on-target activity, representing an important translational milestone that now warrants longer treatment windows, biomarker-anchored endpoints, and more stringent gatekeeping against type I error.

Outside of AD, the most mature exercise-mimetic precedent in neurology comes from fumarates in MS, which leverage the Nrf2-mediated cytoprotective and antioxidative programs that are also induced by physical exercise. In two pivotal phase III trials (NCT00420212 and NCT00451451), dimethyl fumarate (DMF, 240 mg twice daily) significantly reduced the annualized relapse rate, MRI lesion activity, and disability progression relative to placebo, with a safety profile dominated by transient flushing and gastrointestinal symptoms. Subsequent phase IV work (NCT01873417) characterized GI tolerability and pragmatic mitigation strategies. Although MS is primarily immuno-inflammatory rather than primarily degenerative, this body of evidence illustrates that the pharmacological activation of exercise-responsive stress-resilience pathways can achieve regulatory approval and broad clinical uptake when effect sizes are robust and endpoints are disease-relevant.

Taken together, current clinical trials indicate that exercise mimetic pharmacology can achieve measurable target engagement and, in selected conditions such as multiple sclerosis, clinically meaningful efficacy. However, durable benefit will likely require longer treatment windows, biologically enriched populations, and outcome measures that are tightly aligned with the mechanism of action and disease stage.

## Limitations

Despite rapid conceptual advances, most evidence supporting exercise mimetics remains preclinical and relies heavily on rodent models that incompletely capture the genetic, metabolic, and environmental heterogeneity of human disease. This gap is widely recognized as a central barrier to translation, because the multisystem benefits of exercise arise from coordinated adaptations across immune, vascular, metabolic, endocrine, and neural compartments that are difficult to reproduce with any single agent [31].

A first and fundamental limitation is that no intervention can fully mimic the exercise milieu in the foreseeable future [325]. Exercise engages integrated cardiorespiratory dynamics and autonomic regulation, producing coordinated changes in oxygen delivery, substrate flux, thermoregulation, and neuroendocrine tone that are distributed across organs and over time. Moreover, physical activity is embedded in behavior and context, coupling sensorimotor learning, motivation, sleep, and psychosocial factors to neural plasticity and cognitive outcomes. Exercise responses are strongly dependent on temporal structure, including intensity, intermittency, recovery, and long-term training history, creating adaptive trajectories that cannot be readily replicated by constant pharmacological exposure. Exercise mimetics can improve health when used either alone or in combination with exercise. However, pharmacological activation of an exercise-responsive pathway may yield partial molecular signatures without reproducing the integrated physiological adaptations that underpin durable functional outcomes.

Second, the bulk of the evidence comes from preclinical research, and clinical evidence remains uneven. Although some exercise mimetics have entered early-phase trials, the signals in Alzheimer’s disease and mild cognitive impairment have been modest or inconsistent, underscoring the difficulty of bridging peripheral metabolic engagement to meaningful cognitive outcomes in heterogeneous populations. More data are needed in humans, particularly with respect to safety and dosing.

Third, pharmacokinetic, safety, and delivery constraints remain formidable, particularly for central nervous system indications. Achieving therapeutically relevant brain exposure while minimizing systemic toxicity is challenging for many exercise mimetics. Many exercise-responsive pathways are pleiotropic by design, raising concerns about off-target effects and trade-offs when they are chronically manipulated pharmacologically. The long-term activation of metabolic, inflammatory, or mechanotransductive pathways may carry consequences that differ fundamentally from transient, activity-driven engagement, including altered immune tone, vascular instability, or metabolic imbalance. Although innovative delivery platforms, including intranasal carriers and nanomaterial-based systems, show promise in preclinical studies, their scalability, manufacturability, and long-term safety in humans remain insufficiently validated. These considerations are particularly relevant for several frequently cited exercise mimetic candidates. For example, 7,8-DHF is supported by an extensive preclinical literature, yet its development is complicated by pharmacokinetic and formulation limitations that motivate continued optimization rather than immediate clinical extrapolation [326]. Irisin is mechanistically compelling within the muscle-brain axis, but as a peptide mediator it faces practical challenges in standardization, dosing, stability, and delivery that complicate its therapeutic deployment beyond experimental paradigms [327]. Betaine is attractive as a safe nutritional candidate, yet the current evidence for neuroregenerative efficacy is limited and largely indirect, supporting cautious framing until controlled human studies establish target engagement and clinically relevant benefits [320]. Likewise, Piezo1 modulation has emerged as an intriguing mechanotransduction lever, but existing small-molecule tools highlight unresolved issues regarding specificity, context-dependent signaling, and safety, given the roles of Piezo1 across vascular and hematologic systems, arguing against near-term clinical positioning without substantially improved pharmacological precision [328].

Finally, operational and regulatory constraints are nontrivial. Long-term adherence, polypharmacy in older populations, ethical challenges, the cost and complexity of advanced formulations, and uncertainty around personalization workflows all complicate real-world implementation.

## Conclusion and prospects

Collectively, we have synthesized evidence across molecular, physiological, and translational domains to position exercise mimetics as a diverse and evolving class of interventions that engage exercise-responsive biological programs across multiple organ systems. By integrating conceptual foundations, core signaling mechanisms, and biological effects in both normal physiology and major human diseases, we highlight how exercise mimetics operate at the intersection of metabolism, vascular biology, immunity, and neural function. This systems-level perspective underscores that the therapeutic potential of exercise mimetics lies not in reproducing the full complexity of exercise itself, but rather in selectively harnessing specific, biologically actionable components of exercise-induced adaptation. Together, the accumulated evidence reviewed here establishes exercise mimetics as a unifying framework that links fundamental exercise biology to disease-modifying strategies, while setting the stage for a critical evaluation of translational risks, systemic trade-offs, and future opportunities for rational clinical deployment.

Despite rapid conceptual progress, several research gaps warrant focused attention. First, the field still lacks a quantitative map of which components of the exercise response are most amenable to pharmacological modulation without disrupting system-level balance. Second, interindividual variability remains insufficiently resolved. Responsiveness is likely shaped by baseline metabolic state, inflammatory tone, genetics, and comorbidity burden, yet predictive stratification remains rudimentary and is rarely integrated into trial design. Third, the temporal dimension is incompletely understood. Optimal exposure windows, dose scheduling, and the potential for hormetic effects or long-term consequences are poorly defined, impeding the rational design of exercise mimetic administration strategies.

Addressing these gaps will require studies designed not only to test efficacy but also to interrogate mechanism, durability, and systemic integration. For example, to address the off-target effects of exercise mimetics, molecular target identity and binding selectivity can be established early using chemical proteomics to map binding partners across tissues and cell types. Candidate mimetics can then be tested in human-relevant cellular systems representing high-liability tissues or organoids. In addition, trade-off endpoints can be incorporated into preclinical and early clinical studies as key outcomes. Instead of tracking only the intended efficacy pathway, studies could use multi-organ and longitudinal profiling to quantify whether the intervention shifts whole-body homeostasis toward or away from resilience. Finally, because exercise is intrinsically temporally structured, long-term mimetic strategies may need to move toward intermittent or adaptive dosing schemes, supported by longitudinal safety biomarkers and stratified trial designs in real-world, multimorbid populations.

In summary, the promise of exercise mimetics lies in their ability to operationalize aspects of exercise biology for individuals in whom physical activity is insufficient. Key research questions include which pathways can be safely modulated over long timescales, how physiological state and disease stage determine responsiveness, and under what clear criteria combination approaches provide net benefit without amplifying systemic risk. Progress on these questions will advance the development of exercise mimetics as clinically meaningful adjuncts within multimodal therapeutic programs.

## Data Availability

Not applicable.
